# Nanostructure design of 3D printed materials through macromolecular architecture†

**DOI:** 10.1039/d4sc05597g

**Published:** 2024-11-01

**Authors:** Di Wu, Vaibhav Dev, Valentin A. Bobrin, Kenny Lee, Cyrille Boyer

**Affiliations:** a Cluster for Advanced Macromolecular Design, School of Chemical Engineering, University of New South Wales Sydney NSW 2052 Australia; b Australian Centre for Nanomedicine, School of Chemical Engineering, University of New South Wales Sydney NSW 2052 Australia cboyer@unsw.edu.au

## Abstract

Polymerization-induced microphase separation (PIMS) has been previously combined with 3D printing to develop customized nanostructured materials with a wide range of functional applications. In traditional PIMS, monofunctional, linear macromolecular chain transfer agents (macroCTAs) are used to develop macroCTA-*b*-P(monomer-*stat*-crosslinker) networks that self-assemble into unique disordered nanostructures. In this work, we designed a significantly altered network structure by utilizing linear macroCTAs with pendant CTA groups, which provides a novel network upon polymerization (*i.e.*, branched copolymers, [macroCTA-*graft*-[P(monomer-*stat*-crosslinker)]_*n*_]-*b*-P(monomer-*stat*-crosslinker)). Intriguingly, this method leads to the development of alternative disordered morphologies where the internal nanostructure can be precisely controlled. By systematically varying the number of pendant CTA groups, we demonstrate controlled transitions in macroCTA domain continuity, nanodomain size, and phase interface sharpness. These tunable properties translate to adjustable mechanical and swelling behaviors in the resulting 3D printed objects, ultimately enabling the fabrication of smart 4D materials (swelling-induced actuators and temperature-responsive shape-morphing objects). This research significantly expands the design toolbox for 3D printed PIMS materials, providing increased flexibility in the development of advanced materials with specific nanostructures and functionalities.

## Abstract 2

Polymerization-induced microphase separation (PIMS) has been previously combined with 3D printing to develop customized nanostructured materials with a wide range of functional applications.

## Graphical abstract

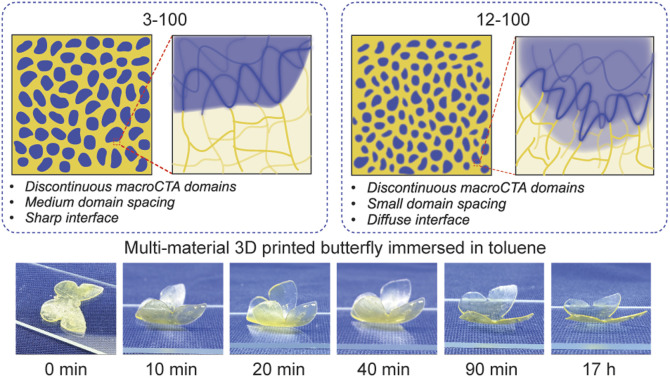

## Introduction

The self-assembly of block copolymers with thermodynamically incompatible blocks has proven to be a highly effective technique for fabricating bulk-state materials with intricate nanostructures.^1,2^ By changing the chemical composition, a rich diversity of ordered morphologies, such as body-centered cubic packed spheres, hexagonally packed cylinders, gyroidal formations, and lamellar structures, have been successfully obtained,^2^ and find wide-ranging applications in catalysis, separations, electronics, and drug delivery.^3,4^ However, achieving these equilibrium morphologies often requires lengthy annealing or other processing steps due to the inherently slow kinetics of block copolymer self-assembly.^2,5^

Alternatively, polymerization-induced microphase separation (PIMS) has been employed to fabricate block copolymer materials with tunable nanodomains and narrow size distribution by a single step procedure.^6^ In a typical PIMS system, a macromolecular chain transfer agent (macroCTA) undergoes chain extension with a monomer and crosslinker to form macroCTA-*b*-(monomer-*stat*-crosslinker) block copolymers.^7^ During this process, the growing block copolymers begin to self-assemble, and kinetically arrested before thermodynamically stable ordered structures, resulting in disordered microphase-separated. The evolution of nanostructures in PIMS systems is affected by many factors including macroCTA molecular weight and loading,^6,8^ monomer selection,^9^ crosslinker concentration,^8^ the use of unreactive homopolymers,^10,11^ and compatibility between macroCTAs.^12^ To date, PIMS has been used to create diverse functional materials for applications such as electrolyte membranes,^13–16^ selective permeable membranes,^17^ drug delivery systems,^18^ catalysts,^19^ and others.^20–22^

Our group successfully developed an ultra-fast and oxygen-tolerant process based on PIMS using RAFT-mediated photochemistry for 3D printing applications.^23^ Fabrication of materials with controlled bicontinuous nanostructures, customized macroscopic geometries and enhanced mechanical properties was achieved using an affordable and commercially available liquid crystal display (LCD) 3D printer.^23^ As the macroCTA plays a critical role in nanostructure evolution in PIMS systems, a number of macroCTA parameters have been explored, including molecular weight,^24^ macroCTA block sequence,^25^ RAFT group selection^26^ and molecular weight distribution.^27^ Despite the current progress in structural control over nanoscale morphology and characteristic size of PIMS materials, most studies have focused on monofunctional linear macroCTAs, and the influence of macroCTA architecture in 3D printing PIMS process has not yet been fully explored.

As an important subclass of polymer architectures, branched polymers are characterized by macromolecular side chains attached to a linear backbone, which encompasses a wide range of structures including graft polymers, star-shaped polymers and hyperbranched polymers.^28^ When applied in a block copolymer context, incompatible branches with the main chain can affect the self-assembly behavior of these copolymers, which can be used to modulate the morphologies,^29,30^ or generate novel morphologies, after microphase separation in bulk.^31–33^ Furthermore, these branched copolymers present interesting mechanical properties in comparison to linear counterparts, such as supersoft materials and high mechanical performances.^34–37^ In 3D printing applications, Shi and coauthors reported unique “phase-inverted” morphologies using star-shaped macroCTAs, highlighting the importance of macroCTA architecture on the development of disordered morphologies.^38^

**Fig. 1:**
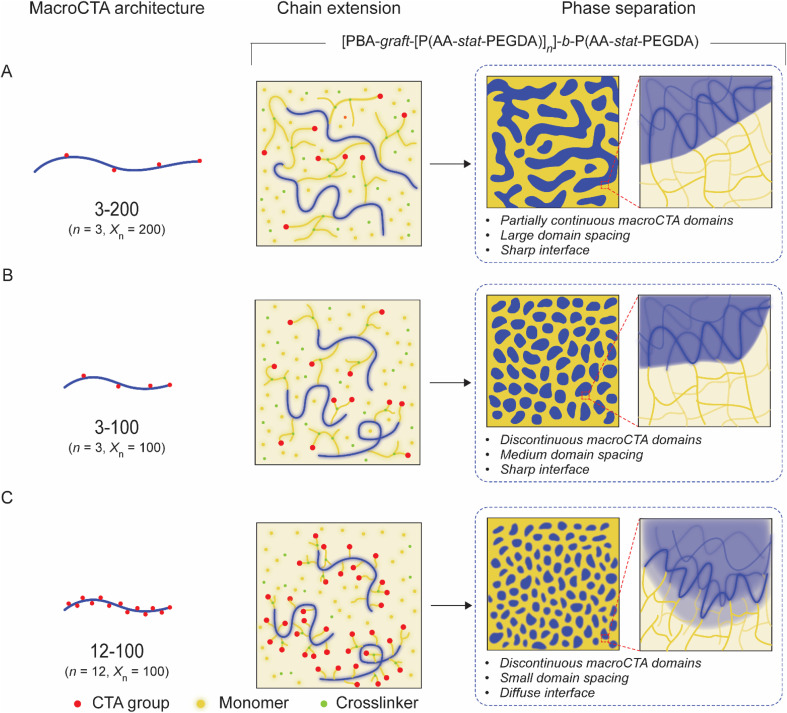
The polymerization-induced microphase separation process and resulting nanostructure of 3D printed materials using various macroCTAs with pendant CTA groups. (A) 3-200 (3 pendant CTA groups on macroCTA backbone with 200 degree of polymerization); (B) 3-100 and (C) 12-100.

Inspired by these previous studies, we have designed a series of multifunctional macroCTAs with pendant CTA groups on the backbone and achieved a novel and reliable approach to nanostructure control (Fig. 1). This design allows for the *in situ* grafting of new polymer blocks onto the macroCTA during 3D printing, enabling the formation of [macroCTA-*graft*-[P(monomer-*stat*-crosslinker)]_*n*_]-*b*-P(monomer-*stat*-crosslinker) polymer networks. By introducing pendant CTA groups and adjusting their number on the linear backbone, we obtained diverse disordered morphologies beyond traditional bicontinuous structures. In particular, the control stems from the ability to tune the continuity of macroCTA domains and regulate domain spacing by adjusting the number of CTA groups and macroCTA molecular weight. We observed a significant impact on the resulting nanostructures, with an upper limit to the number of branching points beyond which phase separation becomes less defined and the influence of phase interfaces becomes increasingly prominent. These tunable nanostructures enable control over bulk mechanical and swelling properties, which we leveraged to manufacture swelling-induced actuators. Additionally, the presence of soft and crosslinked hard domains within these materials were exploited for the fabrication of temperature-responsive shape-morphing objects. The ability to fabricate such materials with complex geometries using a commercial 3D printer highlights the versatility and potential of this approach for applications in soft robotics, biomedical devices, and beyond. This study establishes macroCTAs with pendant CTA groups as a powerful tool for tailoring nanostructure and properties in 3D-printed materials, opening new avenues for the design and fabrication of advanced functional materials.

## Results and discussion

### Synthesis of macroCTAs with pendant CTA groups

To investigate the impact of macroCTAs with pendant CTA groups on 3D printed PIMS materials, a series of macroCTAs with varying numbers of pendant CTA groups were synthesized. Here, a two-step synthesis procedure was utilized, illustrated in Fig. S1 and Table S1 (ESI).† First, thermal RAFT polymerization was performed using *n*-butyl acrylate (BA) and 2-hydroxyethyl acrylate (HEA) as monomers in the presence of 2-(*n*-butylthiocarbonothioylthio) propanoic acid (BTPA) as the RAFT agent to synthesize P(BA_*m*_-*stat*-HEA_*n*_)-CTAs copolymers. Subsequently, utilizing 1-ethyl-3-(3-dimethylaminopropyl)carbodiimide hydrochloride (EDC-HCl) as a coupling agent, pendant lateral trithiocarbonate groups were attached to the P(BA_*m*_-*stat*-HEA_*n*_)-CTAs by esterification of the hydroxyl groups in HEA units with the carboxyl acid group of BTPA. After purification, macroCTAs with pendant CTA groups were obtained and characterized by size-exclusion chromatography (SEC) and proton nuclear magnetic resonance (^1^H NMR, ESI, Fig. S2 and S3†) to determine the number-average molecular weight (*M*_n_), dispersity (*Đ)*, degree of polymerization (*X*_*n*_) and number of pendant CTA groups (*n*), respectively (Table 1). In total, five different macroCTAs were synthesized, with a total target degree of polymerization of either 100 or 200, and either 3, 6 or 12 branching points (Table 1). Successful incorporation of the BTPA into the macroCTA structure was confirmed using NMR spectroscopy, as determined by the disappearance of the CH_2_ α- to the hydroxy group (3.8 ppm), and appearance of a new CH_2_ α- to the ester group (4.3 ppm) after EDC coupling which demonstrated complete esterification of all hydroxy groups in the macroCTA polymers.

**Table 1:** Characteristics of synthesized macroCTAs with pendant CTA groups

MacroCTA^a^	*X*_*n*, BA_ b	*X*_*n*, HEA_ b	*X*_*n*, macroCTA_ b	*n* c	*M*_n,NMR_ d (kg mol^−1^)	*M*_n,SEC_ e (kg mol^−1^)	*Đ*
3-200	196	3	199	3	26.3	27.5	1.16
6-200	190	6	196	6	26.4	26.8	1.15
3-100	98	3	101	3	13.7	14.7	1.13
6-100	95	6	101	6	14.6	14.8	1.16
12-100	92	12	104	12	15.9	16.4	1.16

a MacroCTAs are labelled as “*n-X*_*n*, target_”, where *n* is the number of pendant CTA groups per macroCTA and *X*_*n*, target_ represents the target degree of polymerization of the macroCTA.

b *X*_*n*, BA_ and *X*_*n*, HEA_, are the actual number of BA and HEA units, respectively calculated by NMR; *X*_*n*, macroCTA_ is the actual degree of polymerization of macroCTA. The values were estimated by ^1^H NMR (NMR, ESI, Fig. S2 and S3).

c *n* is the number of pendant CTA groups per macroCTA.

d *M*_n, NMR_ = *X*_*n*, BA_ × MW(BA) + *X*_*n*, HEA_ × MW(HEA) + (*X*_*n*, HEA_ + 1) × MW(BTPA) – *X*_*n*, HEA_ × MW(H_2_O).

e SEC measurements were performed using *N*,*N*-dimethylacetamide (DMAc, containing 0.03% w/v LiBr and 0.05% w/v 2,6-dibutyl-4-methylphenol (BHT)) as an eluent with poly(methyl methacrylate) as calibration standards (SEC).

All synthesized macroCTAs (Table 1) had narrow molecular weight distributions (*Đ* ≤ 1.16) and were classified into two groups according to different target *X*_*n*_ (100 or 200) with the number-average molecular weights in the range of 13.7–16.4 and 26.3–27.5 kg mol^−1^, respectively. The actual degree of polymerization (*X*_*n*, macroCTA_ = *X*_*n*, BA_ + *X*_*n*, HEA_) of macroCTAs estimated by ^1^H NMR spectroscopy exhibited reasonable agreement with the target *X*_*n*_ values (100 or 200). Following post-modification with BTPA and subsequent purification, the number of pendant CTA groups (*n*) per backbone was in accord with the number of HEA units, ranging from 3 to 12 (Table 1, ESI Fig. S2†). To facilitate the comparison across different copolymers, they were categorized based on the number of pendant CTA groups and the target degree of polymerization, *i.e.* BA + HEA units, denoted as 3-200, 6-200, 3-100, 6-100, and 12-100 (Table 1). For instance, 3-200 corresponds to the macroCTA with 3 pendant CTA groups and a target degree of polymerization of 200.

### Formulation of photocurable resins

Having successfully synthesized well-defined macroCTAs with varying number of pendant CTA groups, each macroCTA was incorporated into a 3D printing resin to investigate how their architecture would affect the PIMS process. Photocurable resins were formulated using acrylic acid (AA) and poly(ethylene glycol) diacrylate (PEGDA, average *M*_n_ = 250 g mol^−1^) as the monomer and crosslinker, respectively. This selection was based on their capability to initially solubilize PBA-CTAs, while forming self-assembling incompatible block copolymers after chain extension of PBA-CTAs.^23,39^ Diphenyl(2,4,6-trimethylbenzoyl) phosphine oxide (TPO) was employed as a photoinitiator due to its efficacy in initiating RAFT polymerization under open air conditions, and TPO absorbs photons at a wavelength of 405 nm, which is the operating wavelength of our light-mediated 3D printer.^40–43^ These components were mixed in pre-determined quantities to form homogenous and transparent resins. In all resins, the molar ratio of [AA]/[PEGDA] was fixed at 4/1 and the mass loading of macroCTAs varied between 16.5, 28.2 and 43.9 weight percent (wt%), along with the concentration of TPO at 0.5, 0.7 and 1.0 wt%, respectively, to avoid potential retardation of polymerization from increased CTA group concentration. The resins formulated were denoted using macroCTA code followed by its mass content. For instance, 3-200-16.5 refers to the resin containing 16.5 wt% of 3-200 macroCTA. The compositions of all 15 resins were summarized in Table 2.

**Table 2:** Resin formulations with various branched macroCTAs. The molar ratio of [AA]/[PEGDA] was fixed at 4/1^a^

Resin #	macroCTA	Resin components (wt%)				*X*_p_ of macroCTA
		macroCTA	AA	PEGDA	TPO	
1	3-200	16.5	44.5	38.5	0.5	50
2	6-200					28
3	3-100					25
4	6-100					14
5	12-100					8
6	3-200	28.2	38.1	33.0	0.7	50
7	6-200					28
8	3-100					25
9	6-100					14
10	12-100					8
11	3-200	43.9	29.5	25.6	1.0	50
12	6-200					28
13	3-100					25
14	6-100					14
15	12-100					8

a *X*_p_ corresponds to degree of polymerization divided by the number of total CTA groups. The *X*_p_ value allows comparison of the relative amount of CTA groups between macroCTAs with different degrees of polymerization.

### Photopolymerization kinetics under violet light

**Fig. 2:**
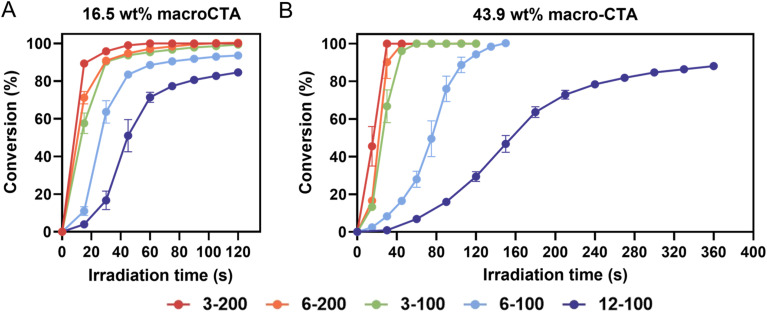
Polymerization kinetics of resins with different macroCTAs and varied mass loading. (A) Resins with 16.5 wt% of macroCTAs (3-200, 6-200, 3-100, 6-100, and 12-100); (B) resins with 43.9 wt% of macroCTAs (3-200, 6-200, 3-100, and 12-100). The kinetics experiments were performed in triplicate. Error bars indicate standard deviation in triplicate measurements. Some error bars fall within the size of the markers.

To ensure reasonable build speeds are attained during the 3D printing process, the photopolymerization kinetics of each resin was investigated by total attenuated total reflectance-Fourier transform infrared (ATR-FTIR) spectroscopy.^24,38^ In these experiments, a 20 μL drop of resin was irradiated under violet light (*λ*_max_ = 405 nm, *I*_0_ = 2 mW cm^−2^) for 120–360 s at 15–30 s intervals, and the ATR-FTIR spectra were recorded between irradiation periods. Vinyl bond conversion was monitored by following the decrease of absorption peak intensity at ≈1620 cm^−1^, which is assigned to the C=C stretching mode of the acrylate groups (AA and PEGDA). Fig. 2 shows typical photopolymerization kinetics of resins containing 16.5 and 43.9 wt% macroCTAs. In Fig. 2A, the conversion of resins formulated with 16.5 wt% macroCTAs increased with the irradiation time, reaching over 80% in 120 s. However, resins containing macroCTAs with 6 or 12 pendant CTA groups exhibited a slower double bond conversion rate. For example, within the group of macroCTAs with *X*_*n*_ of 100 (3-100, 6-100, and 12-100), the vinyl bond conversion of 3-100-16.5 resin was 90.3% at 30 s, while those of 6-100-16.5 and 12-100-16.5 were 63.6% and 16.6%, respectively. This reduction in polymerization rate is attributed to the increase in concentration of CTA groups due to competitive absorption of 405 nm light by the thiocarbonylthio group, which thus results in a lower proportion of TPO photolysis upon irradiation.^26^

With a fixed loading of macroCTA, the concentration of CTA groups in the resins is dependent on the degree of polymerization of the macroCTAs (*X*_*n*, macroCTA_), and the number of CTA groups present in each macroCTA (including pendant CTA groups and the terminal CTA group, denoted as *n* + 1, corresponding to the number of pendant CTA groups plus one (including the terminal CTA group)). To describe the effect of the total CTA concentration on photopolymerization rate more precisely, we calculated the number of repeating units per CTA group (*X*_p_) eqn (1):1
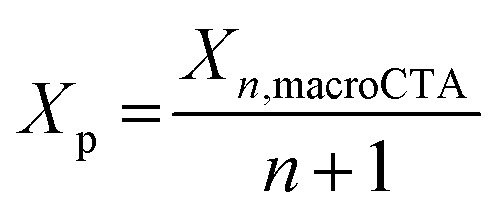


The *X*_p_ value allows comparison of the relative amount of CTA groups between macroCTAs with different degrees of polymerization. As *X*_p_ decreases, the concentration of CTA groups increases, thus resulting in a slower rate of photopolymerization, for example, in the case of 3-200, *X*_p_ = 50 and for 12-100, *X*_p_ = 8 (Table 2). Increasing the weight percentage of macroCTA from 16.5 to 43.9 led to a more pronounced slowing down of polymerization kinetics due to a higher concentration of CTA groups which absorb 405 nm light (Fig. 2B).

### Microscopic morphology and internal nanostructure of 3D printed PIMS materials

Having established that each resin exhibits reasonable polymerization rates, the 15 resins containing macroCTAs with different numbers of pendant CTA groups were applied to a LCD 3D printer equipped with a violet light-emitting diode (LED) light source (Anycubic Photon Mono SE, *λ*_max_ = 405 nm, *I*_0_ = 2 mW cm^−2^) to produce various samples, followed by a 30 min post-cure treatment. The layer thickness and the layer cure time for all resins were set to 100 μm and 60 s, respectively. To assess the formulations' suitability for high-resolution 3D printing, we fabricated star-shaped objects. The printed objects accurately replicated the original designs with good resolution and transparency, indicating the formation of nanodomains (ESI, Fig. S4†).^7^

**Fig. 3:**
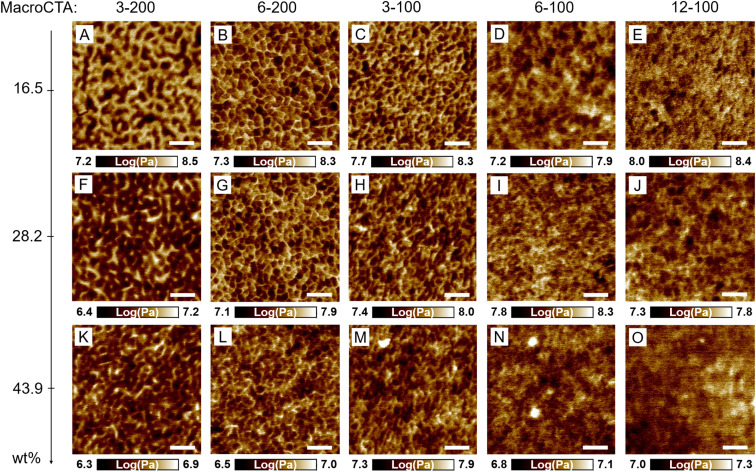
Surface morphologies of 3D printed materials using different loading (16.5, 28.2 and 43.9 wt%) of various branched macroCTAs. (A) 3-200-16.5; (B) 6-200-16.5; (C) 3-100-16.5; (D) 6-100-16.5; (E) 12-100-16.5; (F) 3-200-28.2; (G) 6-200-28.2; (H) 3-100-28.2; (I) 6-100-28.2; (J) 12-100-28.2; (K) 3-200-43.9; (L) 6-200-43.9; (M) 3-100-43.9; (N) 6-100-43.9; (O) 12-100-43.9. The AFM images were obtained with PeakForce quantitative nanomechanics (QNM) mode to acquire DMT modulus map images. Dark area refers to soft PBA macroCTA domains and light area refers to hard *net*-P(AA-*stat*-PEGDA) domains. Scale bars are 60 nm.

3D printed square prism samples (*l* × *w* × *t* = 8 × 8 × 2 mm) were analyzed using atomic force microscopy (AFM) to visualize any nanoscale morphologies that may arise from the photoinitiated PIMS process. PeakForce tapping mode was employed to distinguish between domains with different modulus. In these block copolymer systems, the macroCTA generates soft domains with a low modulus, attributed to low *T*_g_ of PBA, while the crosslinked *net*-P(AA-*stat*-PEGDA) network results in hard domains with a high modulus.^23,24^Fig. 3 shows some representative AFM images, while lower magnification AFM images are exhibited in ESI, Fig. S5–S7.†

Based on the AFM images (Fig. 3), disordered microphase separated morphologies were observed for all samples with varying degrees of continuity in all cases. Considering the surface morphologies of samples made with 16.5 wt% 200 *X*_*n*_ macroCTA, the 3-200-16.5 sample appeared disordered with fully continuous *net*-P(AA-*stat*-PEGDA) domain. Comparatively, the PBA were only partially continuous and had an elongated domain appearance (Fig. 3A). Increasing the number of pendant CTA groups to 6 (6-200-16.5) resulted in a dramatic change in the morphology of the PBA domains, which appeared as isolated discontinuous globules dispersed in the hard *net*-P(AA-*stat*-PEGDA) domain (Fig. 3B). In addition to an effect on the continuity of the PBA phase, the *net*-P(AA-*stat*-PEGDA) domain width (*D*_net_) was also heavily affected by the number of pendant CTA groups on macroCTA. Interestingly, when the number of pendant CTA groups was increased, the *net*-P(AA-*stat*-PEGDA) domain size was reduced from 11 to 7 nm while the macroCTA domain width (*D*_m_) remained relatively constant (∼11–12 nm) for the 16.5 wt% samples (Table 3 and Fig. S8†). This result is expected, as an increased number of branching points lowers the degree of polymerization in the *net*-P(AA-*stat*-PEGDA) phase thus reducing its associated domain spacing, while the macroCTA domain width is not affected due to the fixed degree of polymerization of the macroCTA.

**Table 3:** Nanostructure characteristics of 3D printed PIMS materials

MacroCTA		PBA domain^a^		*net*-P(AA-*stat*-PEGDA) domain^a^	*χN*_p_ b	*D*_m_ c (nm)	*d*_AFM_ c (nm)	*D*_net_ c (nm)	*d*_SAXS_ d (nm)	*d*_TS_ e (nm)
wt%	Type	Continuity	Appearance	Continuity						
16.5	3-200	Partially continuous	Elongated	Continuous	180	12	23	11	21	19
	6-200	Discontinuous	Globular	Continuous	103	11	18	7	14	13
	3-100	Discontinuous	Globular	Continuous	94	10	16	6	13	12
	6-100	Discontinuous	Blur	Continuous	56	—	—	—	9	8
	12-100	Discontinuous	Blur	Continuous	33	—	—	—	7	6
28.2	3-200	Continuous	Bicontinuous	Continuous	103	12	21	9	19	18
	6-200	Discontinuous	Globular	Continuous	59	11	17	6	13	12
	3-100	Discontinuous	Globular	Continuous	53	9	15	6	12	11
	6-100	Discontinuous	Blur	Continuous	32	—	—	—	8	8
	12-100	Discontinuous	Blur	Continuous	19	—	—	—	7	6
43.9	3-200	Continuous	Bicontinuous	Continuous	64	11	18	7	17	17
	6-200	Partially continuous	Elongated	Continuous	36	10	14	4	12	12
	3-100	Partially continuous	Elongated	Continuous	33	—	—	—	11	11
	6-100	Discontinuous	Blur	Continuous	20	—	—	—	8	8
	12-100	Discontinuous	Blur	Continuous	11	—	—	—	7	6

a Morphology of 3D printed PIMS materials determined by AFM.

b Segregation strength (see details in ESI, Note S1 and Table S2).

c PBA domain width (*D*_m_), center-to-center domain spacing (*d*_AFM_) and *net*-P(AA-*stat*-PEGDA) domain width (*D*_net_) determined by AFM (Fig. S8–S11).

d Domain spacing (*d*_SAXS_) determined by SAXS.

e Domain spacing (*d*_TS_) calculated using T–S fitting (see details in ESI, Table S3).

For all 200 *X*_*n*_ macroCTA samples, increasing the macroCTA concentration led to an increase in PBA continuity for samples with both 3 and 6 pendant CTA groups. For example, for samples using the 3-200 macroCTA, the PBA domain appeared partially continuous and elongated for the 16.5 wt% sample, but appeared significantly more continuous in 28.2 and 43.9 wt% samples (Fig. 3A, F and K). A similar trend of increased continuity with increasing macroCTA concentration is observed for the 6-200 samples, which appeared globular at 16.5 wt% and 28.2 wt% loading, and continuous at 43.9 wt% (Fig. 3B, G and L). Notably, the *net*-P(AA-*stat*-PEGDA) phase appeared continuous in all 200 *X*_*n*_ samples, which indicates that the increased branching points primarily affects the continuity in the PBA phase. Similar to the effect of the number of branching points, increasing the macroCTA concentration increases the concentration of CTA groups while reducing the total mass fraction of AA and PEGDA in the original resins, with both factors expected to reduce the *net*-P(AA-*stat*-PEGDA) domain size. This is reflected in the AFM measurements (Table 3), where the PBA domain width was similar in all cases regardless of concentration or number of branching points (10–12 nm), while the *net*-P(AA-*stat*-PEGDA) domain width was significantly reduced upon increasing the macroCTA concentration (11, 9, and 7 nm for the 3-200 group and 7, 6, and 4 nm for the 6-200 group at 16.5, 28.2 and 43.9 wt% respectively).

The restriction of continuity in the macroCTA domains simply by incorporating a few branching points strongly contrasts typical PIMS systems,^6,23^ which contain only one chain extension site. In the macroCTAs used in this work, the additional chain extension sites led to an increased number of P(AA-*stat*-PEGDA) branches, and consequently a decrease in the average P(AA-*stat*-PEGDA) chain length. The presence of P(AA-*stat*-PEGDA) branches surrounding the PBA units on the macroCTA backbone consequently frustrates the assembly of PBA domains, resulting in reduced macroCTA domain continuity, and inhibits the formation of a typical bicontinuous morphology. However, this effect is subdued upon further increasing the macroCTA concentration due to an increased volume fraction of PBA, where a higher volume fraction of PBA allows the macroCTA domains to assemble into continuous channels.

While the nanostructure of 3-100 *X*_*n*_ macroCTA samples was readily observable using AFM, further increasing the number of branching points (6-100 and 12-100 samples) resulted in less defined structures with significantly blurred phase boundaries (Fig. 3D, I and N for 6-100; Fig. 3E, J and O for 12-100). The poorly defined morphologies in the 6-100 and 12-100 groups may be attributed to weaker phase segregation. In block copolymer self-assembly, the segregation strength (*χN*) describes the thermodynamic driving force for microphase separation between incompatible blocks, where *χ* is the interaction parameter of different blocks and *N* is the total degree of polymerization of the block copolymer.^8,44,45^ In this system (assuming negligible contribution from esterified BTPA units), the interaction parameters (*χ*) for PBA-*b*-P(AA*-stat*-PEGDA) block copolymers was estimated to be 0.505 at 25 °C (ref. 24) (ESI, Note S1†), indicating thermodynamically incompatibility between PBA and P(AA*-stat*-PEGDA) blocks.^1^ For branched block copolymer formed in this system, the average degree of polymerization per CTA group (*N*_p_) of branched block copolymers was estimated by the following eqn (2):2
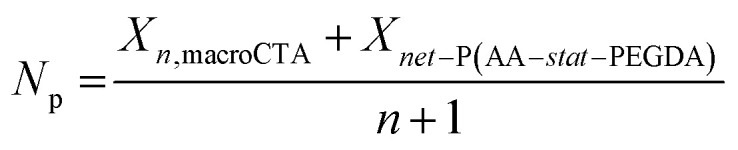
where *X*_*net-*P(AA*-stat-*PEGDA)_ is the degree of polymerization of *net*-P(AA-*stat*-PEGDA) network.

The calculated *χN*_p_ are summarized in Table 3 (see details in ESI, Table S2†). Increasing the number of CTA groups per polymer chain significantly reduces the segregation strength due to a lower degree of polymerization in the *net*-P(AA-*stat*-PEGDA) block. For example, the *χN*_p_ for 12-100-43.9 was 11, approaching the limit value for disorder–order transition.^2,46^

Ultimately, these results indicate that the presence of multiple P(AA-*stat*-PEGDA) branches frustrates the ability of the PBA domains to align into continuous channels, while the *net*-P(AA-*stat*-PEGDA) remains continuous in all cases. Interestingly, the formation of multiple branches does not appear to affect the macroCTA domain width, and instead only results in a reduction of the *net-*P(AA-*stat*-PEGDA) domain width. Upon significantly increasing the number of pendant CTA groups to 12 at a macroCTA *X*_*n*_ of 100, a significantly blurred interface between domains is observed, which is attributed to two factors: a weaker segregation strength that approaches the lower limit for a disorder–order transition, and an increased frustration of self-assembly in the PBA domain due to the increased occurrence of incompatible branches.

**Fig. 4:**
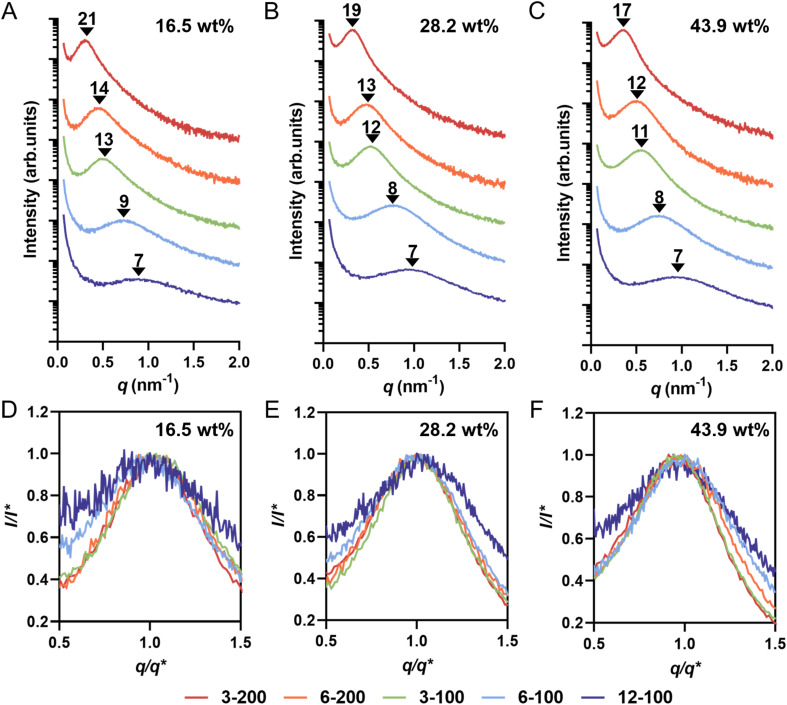
(A)–(C) SAXS profiles and domain spacing (*d*_SAXS_) values of 3D printed materials prepared by different loading of macroCTAs with various pendant CTA groups: (A) 16.5 wt%, (B) 28.2 wt%; and (C) 43.9 wt% of macroCTA. (D)–(F) Comparison of normalized peak broadness of SAXS profiles based on the principal peak position (q*) and intensity (I*) for 3D printed materials prepared by different loading of macroCTAs with various pendant CTA groups: (D) 16.5 wt%, (E) 28.2 wt%; and (F) 43.9 wt% of macroCTA.

Small-angle X-ray scattering (SAXS) experiments were conducted on 3D printed rectangular prism samples with dimensions of *l* × *w* × *t* = 6 × 6 × 0.2 mm to gain further insight into the internal nanostructure of 3D printed materials. Fig. 4A–C shows the SAXS profiles and corresponding domain spacing (*d*_SAXS_) values of samples prepared with different macroCTAs at three different loadings (16.5, 28.2 and 43.9 wt%). All samples exhibited a single scattering peak, which is characteristic of a disordered microphase-separation state as typically observed in PIMS processes. Notably, the 6-100 and 12-100 groups, which had unclear morphologies in AFM images (Fig. 3D, I and N for 6-100; Fig. 3E, J and O for 12-100), showed a broad peak in their SAXS profiles, indicating some internal nanostructure formation and microphase separation.

Across all formulations, increasing the number of pendant CTA groups on the macroCTA backbone led to a shift of the SAXS scattering peak to a larger *q* value, signifying a decrease in domain spacing calculated from SAXS (*d*_SAXS_). For example, in the group with 16.5 wt% macroCTA with *X*_*n*_ of 200 (*i.e.*, 3-200 and 6-200), *d*_SAXS_ decreased from 21 nm to 14 nm (Fig. 4A). Similarly, for the 16.5 wt% macroCTA group with *X*_*n*_ of 100 (*i.e.*, 3-100, 6-100 and 12-100), *d*_SAXS_ values consistently decreased from 13 to 9 and then to 7 nm (Fig. 4A). At a fixed macroCTA composition of 16.5 wt%, *d*_SAXS_ values correlated with decreasing *X*_p_ across all five macroCTAs (Table 2). This trend was also observed in the 28.2 wt% (Fig. 4B) and 43.9 wt% (Fig. 4C) macroCTA formulations. The decrease in *d*_SAXS_ is attributed to the reduced P(AA-*stat*-PEGDA) branch chain length with increasing pendant CTA groups. Pleasingly, the SAXS domain spacings (*d*_SAXS_) values closely matched the center-to-center domain spacings measured by AFM (*d*_AFM_) which supports the validity of both measurements (Table 3).

Additionally, to investigate the effect of the polymerization kinetics on nanostructure formation, *in situ* SAXS experiments were conducted using 3-100-28.2 resin as model, exposed to violet light (405 nm, 2 mW cm^−2^) for varying durations (0, 10, 20 30 and 60 s). As shown in ESI, Fig. S12A,† the scattering peak positions and SAXS profiles for these different time points did not change, in the exception of progressively increasing scattering intensity with irradiation time indicated that the characteristic domain spacing remained constant throughout the polymerization process, aligning with previous findings.^6,47,48^ Furthermore, variations in polymerization rates, caused by different light intensities (1 and 2 mW cm^−2^), had minimal impact on the formation of nanostructures according to the SAXS profiles (ESI, Fig. S12B†). Similarly, annealing samples (overnight at 120 °C) did not affect the SAXS profiles (ESI, Fig. S12C†).

**Fig. 5:**
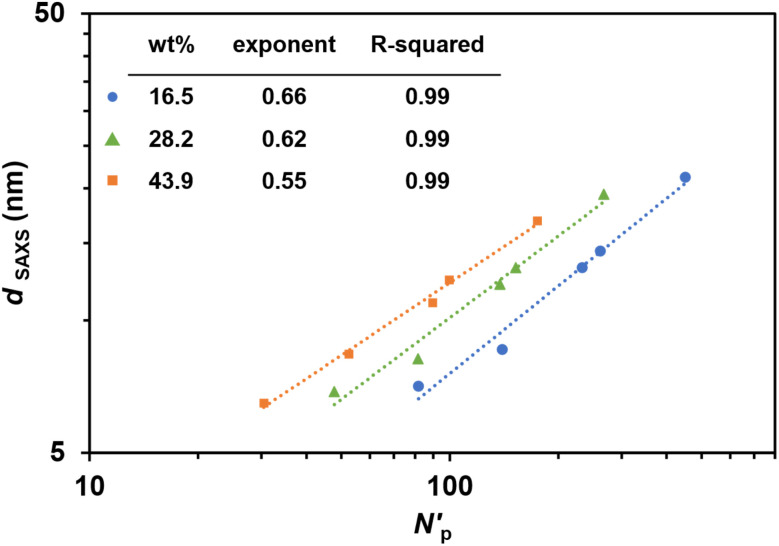
Power law regression lines for domain spacing of 3D printed PIMS materials. log–log plot of domain spacing (*d*_SAXS_) as a function of normalized total degree of polymerization per CTA group (*N*′_p_). *d*_SAXS_ was determined from SAXS. *N*′_p_ was normalized based on a common monomer reference volume (118 Å^3^). Scaling exponents and *R*-squared values are shown in the inset table.

In a typical PIMS system, the characteristic domain spacing is constrained by the radius of gyration of the diblock copolymer, which can be effectively tuned by modulating the macroCTA molecular weight.^24,47^ However, in this work using macroCTAs with pendant CTA groups, domain spacing can also be adjusted by varying the number of pendant CTA groups on macroCTA backbone while maintaining macroCTA molecular weight. Controlling nanoscale domain spacing of microphase-separated copolymer materials is crucial for potential applications, as nanostructure influences mechanical and physiochemical properties (such as swelling).^24,38^ In previous microphase-separated systems, domain spacing (*d*) has been observed to be proportional to the total degree of polymerization of the block copolymer (*N*) according to a power law *d*–*N*^a^.^49^ Based on block copolymer theory, block copolymer chains exist in a perturbated, more stretched conformation with *d*–*N*^2/3^ in the strong segregation limit, while in unperturbed conformation with *d*–*N*^1/2^ in the weak segregation limit^49^ Considering the branched copolymer network formed in this 3D printed PIMS system, *d*_SAXS_ as function of the total volumetric degree of polymerization per CTA group (*N*′_p_) was fitted with a power law model (Fig. 5). Interestingly, regardless of the number of pendant CTA groups on the macroCTA backbone, the obtained power exponents ranged from 1/2 to 2/3 with loadings of macroCTA.^44^ Thus, the derived power law provides a method for predicting size of nanostructural feature sizes in 3D printed PIMS materials employing macroCTAs with pendant CTA groups.

To further analyze the microphase-separated nanostructures of 3D printed materials, the SAXS curves were fitted using the Teubner–Strey (T–S) model.^50–52^ Structural parameters, including domain spacing (*d*_TS_), correlation length (*ξ*), and amphiphilicity factor (*f*_a_), were obtained from the T–S model fitting. The fitted SAXS patterns are presented in ESI, Fig. S13–S15† and the extracted structural parameters are listed in Table 3 and ESI, Table S3.†*d*_TS_ characterizes the periodic spacing between domains. The domain spacing values determined by the T–S model fitting (*d*_TS_) closely aligned with those obtained from SAXS profiles (*d*_SAXS_), confirming the suitability of the T–S model for our system (Table 3). *ξ* reflects the spatial coherence of the interfaces, and the ratio *ξ*/*d*_TS_ serves as a measure of domain size polydispersity.^53^ In both groups with *X*_*n*_ of 100 (3-100, 6-100 and 12-100) and 200 (3-200 and 6-200), a decrease in the *ξ*/*d*_TS_ values was observed with increasing pendant CTA groups, suggesting greater polydispersity in domain size. For example, at 16.5 wt% macroCTA loading, the *ξ*/*d*_TS_ ratio decreased from 0.47 for 3-200 to 0.41 for 6-200, and from 0.45 for 3-100 to 0.33 for 12-100. In the T–S model, *f*_a_ characterizes the segregation strength at the interfaces. When *f*_a_ > 0, the material is weakly-structured; −1 < *f*_a_ < 0 corresponds to well-structured materials; *f*_a_ = −1 corresponds to interfacial segregation strengths for ordered lamellar structures.^44^ At a fixed degree of polymerization of macroCTA (100 or 200), the *f*_a_ values increased from −0.8 to −0.6 as the number of pendant CTA groups increased. This trend suggests a weakening of phase separation and poorer phase interfaces with increasing pendant CTA groups, particularly for 6-100 and 12-100 samples. These results are consistent with the broader peaks normalized SAXS profiles (Fig. 4D–F), further supported by the increased peak widths at 60% maximum and the decreased Porod exponents (ESI, Table S3†), indicating a transition from a relatively sharp interface to more diffuse one with increasing branching points.^8,54^

In microphase-separated block copolymer systems, an interfacial layer exists between the two incompatible domains where A blocks and B blocks are mixed.^55–57^ In contrast to the diblock copolymer system, the presence of *net*-P(AA-*stat*-PEGDA) branches reduces segregation strength between blocks and physically frustrates the overall microphase separation process, leading to weaker microphase separation and a broader interfacial layer range, which is indicated in the breadth of the SAXS profiles. The normalized SAXS profiles (Fig. 4D–F) reveal significantly broader peaks for samples prepared with 6-100 and 12-100 compared to other samples, despite having small domain spacings (7–9 nm). This contrasts previous reports using linear macroCTAs, where samples prepared by smaller macroCTAs typically produce sharper SAXS peaks.^24^ This suggests that while pendant CTA groups can control the characteristic domain spacing by reducing the degree of polymerization of the P(AA-*stat*-PEGDA) phase, the resulting morphology is less well-defined, with a broader interfacial region. The broader domain interfaces identified by T-S modeling may also explain the inability to visualize PIMS morphology *via* AFM in materials 3D printed using 6-100 and 12-100 macroCTAs (Fig. 3).

### Bulk properties of 3D printed PIMS polymers

**Fig. 6:**
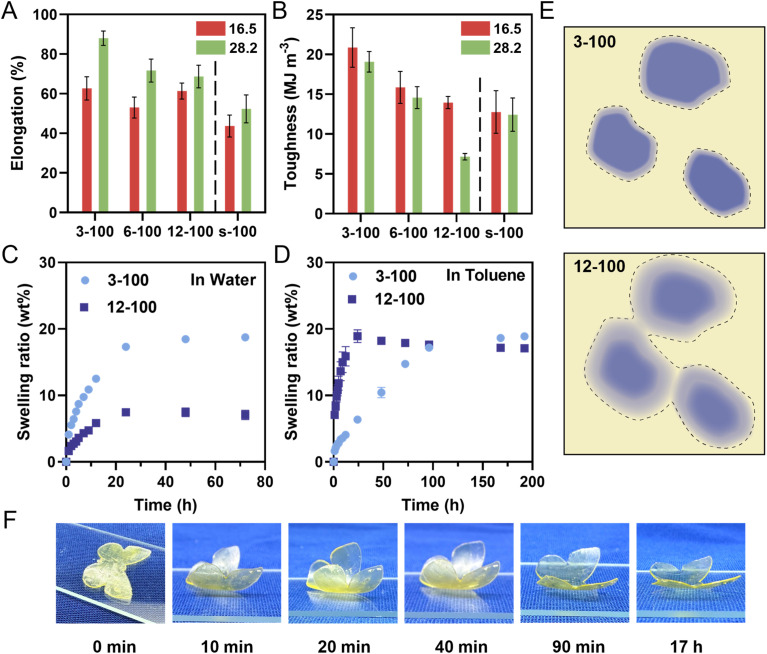
(A) and (B) Mechanical properties of 3D printed PIMS materials using two loadings (16.5 and 28.2 wt%) of macroCTAs with pendant CTA groups (3-100, 6-100 and 12-100) and corresponding non-PIMS materials (s-100) by tensile testing. (A) Elongation at break; (B) toughness. Materials were 3D printed using a molar ratio of [AA]/[PEGDA] = 4/1 at a varied loading of macroCTAs or mixtures of BA and RAFT agent (BTPA) and the dash line separated PIMS and non-PIMS materials error bars indicate standard deviation in at least triplicate measurements. (C) and (D) Swelling ratio (wt%) of 3D printed PIMS materials using 28.2 wt% of 3-100 and 12-100 in different solvents. (C) Swelling ratio in water. (D) Swelling ratio in toluene. Error bars indicate standard deviation in duplicate measurements. Some error bars fall within the size of the markers. (E) Interface diagram of PIMS system using 3-100 and 12-100 macroCTA (the dotted line marks the expected swelling range in toluene). (F) Swelling-induced actuation in toluene of a multi-material butterfly 3D printed using two kinds of resins, 3-100-28.2 and 12-100-28.2 (side view).

To investigate the impact of internal nanostructures on mechanical properties of 3D printed materials, we conducted tensile tests on PIMS samples and corresponding chemically analogous non-PIMS terpolymer. Dumbbell-shaped samples were 3D printed using a molar ratio of [AA]/[PEGDA] = 4/1, with 16.5 and 28.2 wt% of three kinds of macroCTAs (3-100, 6-100 and 12-100) or mixtures of BA, AA, PEGDA and RAFT agent (BTPA) (denoted as s-100). The molar ratio of BA to BTPA in the non-PIMS resins was set at 100/1 (ESI, Table S4†), yielding non-microphase separated statistical copolymers (*net*-P(BA-*stat*-AA-*stat*-PEGDA) with similar composition. Fig. 6A and B, S16 and Table S5 (ESI†) display the tensile properties of PIMS materials and corresponding non-PIMS materials. Elongation at break of materials 3D printed with 16.5 wt% of 3-100, 6-100 and 12-100 were 62.7, 53.0 and 61.3%, respectively, which are greater than 43.7% for the s-100 statistical copolymer. At 28.2 wt% loading, elongation increased for all samples compared to 16.5 wt% loading but decreased with the inclusion of more branching points (from 3-100 to 12-100), consistently higher than that of s-100. This demonstrates that the nanostructure resulting from microphase separation improves material ductility due to efficient stress dissipation between soft and hard domains.^24,58,59^ The effect of pendant CTA groups on mechanical properties was also evident in the tensile stress at break values and Young's modulus (Table S5†). At both 16.5 and 28.2 wt% macroCTA loadings, the Young's modulus was significantly lower for 12-100 samples compared to all other samples. Toughness, an important indicator of a material's ability to absorb a mechanical stress, decreased with increasing pendant CTA groups on the macroCTA backbone (Fig. 6B). For example, the average toughness of 3-100-16.5 was 20.9 MJ m^−3^, while those of 6-100-16.5 and 12-100-16.5 decreased to 15.9 and 14.0 MJ m^−3^, respectively. The non-phase-separated sample (s-100-15.6) exhibited the lowest toughness of 12.8 MJ m^−3^.

Overall, these results highlight the impact of microphase separation and pendant CTA group number on the mechanical properties of 3D printed materials. Increasing pendant CTA groups generally reduces elongation at break, tensile stress, Young's modulus, and toughness. To be specific, the gradual decrease in mechanical properties with increasing pendant CTA groups can be attributed to two points. Firstly, using macroCTAs with a higher number of branching points resulted in less continuous domains and reduced stress dissipation efficiency. Secondly, pendant CTA groups on macroCTA backbone decrease the average chain length in crosslinked phase, leading to lower segregation strength (Table 3) and less-defined phase interfaces as noted by a lower *f*_a_ values (ESI, Table S3†).^60–62^ Overall, pendant CTA groups on macroCTA backbone offer 3D printed materials a flexible means to tune mechanical properties, which is beneficial for various structural applications requiring precise control over mechanical characteristics.

To further investigate the properties of PIMS materials prepared with varying pendant CTA groups, the swelling behavior of 3D printed square prism materials (*l* × *w* × *t* = 8 × 8 × 2 mm) using a fixed 28.2 wt% loading of 3-100 and 12-100 was investigated in both water and toluene. Given the hydrophobic nature of PBA phase and the hydrophilic *net*-P(AA-*stat*-PEGDA) phase, water preferentially swells the hydrophilic crosslinked *net*-P(AA-*stat*-PEGDA) domains, while toluene preferentially swells the hydrophobic PBA domains. In water, the swelling ratios of 3-100-28.2 and 12-100-28.2 printed samples plateaued at 18.7 and 7.1 wt%, respectively, after 72 hours (Fig. 6C). Interestingly, their counterparts in toluene exhibited contrasting swelling behaviors (Fig. 6D). Here, the swelling ratio of 12-100-28.2 increased significantly faster than 3-100-28.2 in the first 24 hours, and then reached a plateau at 17.1 wt% after 192 hours. In contrast, the swelling ratio of 3-100-28.2 increased at a slower rate, eventually reaching equilibrium around 18.9 wt%, which was slightly higher than 17.1 wt% for 12-100-28.2.

In conventional materials prepared by diblock copolymers, the swelling ratio of a fixed material composition primarily depends on the domain volume and continuity.^25,38^ However, in our specific system, the phase separation is less defined with diffuse interfaces; therefore, the interface may play a significant role in the swelling behavior of these systems. To explain the unprecedentedly high uptake of toluene in the 12-100 sample, we hypothesized that while the PBA domains may not appear continuous under AFM, the interfacial regions could possess some continuity facilitating swelling and mass transfer throughout the bulk material. An illustration of domain interface continuity is represented in Fig. 6E.

To test this assumption, a material with an approximate composition of a 1 : 1 [BA]:[AA + PEGDA] interface was 3D printed using a statistical copolymers using a mixture of BA, AA, PEGDA monomers with BTPA (the exact molar ratio of [BA]:[AA + PEGDA][BTPA] = 40 : 40 : 1, Table S6†): and subsequently, the swelling behavior was determined (Fig. S17†). Interestingly, the materials obtained by statistical copolymerization showed significant swelling in toluene (24.9%), but negligible swelling in water. This suggests that a statistical PBA-*stat*-PAA-*stat*-PEGDA copolymer is more hydrophobic than hydrophilic and a diffuse interface containing both polymers obtained in 12-100-28.2 is capable of swelling in toluene, but less so in water. Consequently, the 12-100-28.2 sample, with a diffuse and likely continuous interfacial region, showed a faster swelling rate in toluene. Ultimately, both materials ultimately reached a similar equilibrium swelling ratio in toluene, as expected due to equal PBA volume fractions in both samples.

The swelling behavior difference in toluene, based on pendant CTA group number, offers a unique design strategy for the fabrication of 4D materials, which was not previously reported.^63^ As a proof of concept, a butterfly-shaped actuator was fabricated by successively printing with resins containing macroCTAs with 3 and 12 pendant CTA groups (ESI, Fig. S18A†). When immersed in toluene, the lower part of the sample prepared using 12-100-28.2 swelled more rapidly than the upper part which was prepared using 3-100-28.2 resin, causing the wings to rapidly bend to an angle of 42.4° within 10 minutes (Fig. 6F and S18B†), with a maximum bending angle of 47.7° observed after 20 minutes. This bending is attributed to the increased uptake of the lower part during the early stages of immersion. After 20 minutes, the bending angle gradually decreased to 44.3° at 40 minutes, as the 12-100-28.2 layers reached their maximum swelling capacity while the upper 3-100-28.2 layers continued to absorb toluene. After 17 hours, the butterfly wings returned to their original configuration, achieving a small angle of 7.3° due to similar volume uptake in both the upper and lower layers (Fig. S18B†). This effect occurs because the 3-100-28.2 and 12-100-28.2 formulations ultimately demonstrate similar equilibrium swelling ratios after prolonged immersion in toluene.

Beyond the typical swelling-driven behavior of 4D materials,^63^ the PIMS system presented here, with its distinct soft and crosslinked hard domains, exhibits promising potential as shape memory polymers (SMPs).^64^ To investigate this, we explored the temperature-responsive shape memory properties of these materials. The glass transition temperature (*T*_g_) of the sample prepared using 3-100-28.2 resin was determined to be approximately 46 °C by differential scanning calorimetry (DSC) (ESI, Fig. S19†). A dumbbell-shaped 3-100-28.2 sample was printed and placed at 70 °C to soften the materials, enabling to be deformed by force to create a specific shape ESI.† After rapid cooling down to room temperature, the deformed shape was fixed. Reheating the sample to 70 °C resulted in rapid and complete recovery of the original shape within one minute (see ESI, Video 1†). This shape memory cycle can be repeated multiple times with consistent and reliable performance, demonstrating the robust temperature-responsive shape memory capabilities of this PIMS-derived material.

### Macroscopic fabrication of complex shaped objects

**Fig. 7:**
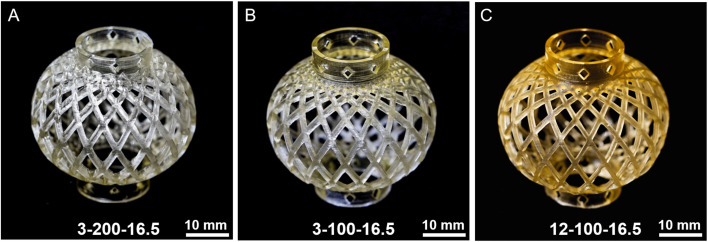
3D printed Chinese lantern models using three types of macroCTA with pendant CTA groups at 16.5 wt% loading. (A) 3-200; (B) 3-100; (C) 12-100. The objects were 3D printed at layer cure time of 40 s with a layer thickness of 100 μm with a molar ratio of [AA]/[PEGDA] = 4/1 in resins.

To demonstrate the capacity to fabricate complex geometries using our resins, Chinese lantern models were 3D printed using a commercially available LCD 3D printer. Fig. 7A–C shows three successfully printed lanterns using 16.5 wt% of 3-200, 3-100, and 12-100 macroCTAs, exhibiting high fidelity compared to the digital model (ESI, Fig. S21†). All printed objects accurately reproduced the external geometry and complex internal features, including diamond-shaped holes in the lower and upper parts, and interleaving square lines in the middle body. A progressive increase in yellow color intensity was observed from 3-200-16.5 to 3-100-16.5 and 12-100-16.5, which is attributed to higher concentrations of trithiocarbonate groups in each respective resin. Furthermore, a slight overcuring was observed for 3-200-16.5 (junction of the globular and annular parts), which was reduced when the number of pendant CTA groups increased (3-100-16.5 and 12-100-16.5). This increase in print resolution in resins with a higher concentration of 405 nm absorbing thiocarbonylthio groups has been previously demonstrated.^40^ This enhancement occurs because these groups prevent light scattering, thus improving print resolution of all axes. Overall, the PIMS resin system studied here demonstrated precise and rapid fabrication of geometrically intricate objects with internal nanostructures, thus enabling the control of material features on multiple length scales.

## Conclusion

This study pioneers the use of macroCTAs with pendant CTA groups for 3D printing PIMS polymer materials. With the branching points provided by pendant CTA groups, a novel branched block copolymer structure ([PBA-*graft*-P[(AA-*stat*-PEGDA)]_*n*_]-*b*-P(AA-*stat*-PEGDA), where *n* = 3, 6 or 12) was formed upon photopolymerization 3D printing, differing from previous reports using monofunctional, linear-macroCTAs. The presence of branching points was found to significantly alter the self-assembly process, which heavily influenced the nano-scale structure and functional properties of the resultant disordered microphase-separated materials. Interestingly, the PBA domain continuity and appearance were significantly affected by the number of pendant CTA groups on the macroCTA backbone. As the number of branching points were increased, the domain size of the *net*-P(AA-*stat*-PEGDA) was also reduced, which was accompanied by a transition from sharp to diffuse interfaces between PBA and *net*-(P(AA-*stat*-PEGDA) phases. Notably, the domain spacing of microphase-separated nanostructures was tuned by manipulating the number of pendant CTA groups while maintaining a constant degree of polymerization of macroCTAs. The scaling behavior of the domain spacing was strongly predictable, following a power law function with the normalized degree of polymerization of the block copolymer per CTA (*N*′_p_).

This work provides an avenue for controlling the morphology and properties of 3D printed polymer materials, as demonstrated by swelling and mechanical tests. Microphase-separated 3D printed materials exhibited increased elongation and toughness compared to non-PIMS materials. Additionally, the swelling kinetics of these PIMS materials were tuned by the number of pendant CTA groups, which was utilized in the fabrication of 4D printed object. In addition, the presence of soft and crosslinked hard domains within materials have been successfully exploited for the preparation of temperature-responsive shape-morphing objects. The ability to fabricate customized complex geometries was also demonstrated by precisely reproducing a digital Chinese lantern model using a commercially available 3D printer, which demonstrates control over material structuration across multiple length scales. Overall, the 3D printed PIMS system developed in this work not only expands the toolbox for controlling the nanostructure of 3D printed polymer materials, but also opens the possibilities for advanced material preparation, with potential applications in energy storage, structural components, and catalysis.

## Notation

The following symbols and notations are used throughout the main text:

*M*_n_
: Number-average molecular weight of macroCTAs
Đ
: Dispersity of macroCTAs
*X*_*n*_
: Degree of polymerization of macroCTAs
n
: Number of pendant CTA groups per macroCTA backbone
*X*_p_
: Degree of polymerization per CTA group of macroCTAs
*D*_m_
: PBA domain width determined by AFM
*d*_AFM_
: Center-to-center domain spacing determined by AFM
*D*_net_
: *net*-P(AA-*stat*-PEGDA) domain width determined by AFM
χ
: Interaction parameter of different blocks
N
: Total degree of polymerization of the block copolymer
*X*_*net-*P(AA*-stat-*PEGDA)_
: Degree of polymerization of *net*-P(AA-*stat*-PEGDA) network
*N*_p_
: Total degree of polymerization per CTA group of the block copolymer
*N′*_p_
: Total volumetric degree of polymerization per CTA group of the block copolymer
*d*_SAXS_
: Domain spacing determined by SAXS
q*: Peak position of the principal peak in SAXS profiles
I*: Peak intensity of the principal peak in SAXS profiles
*d*_TS_
: Domain spacing determined by T–S fitting
ξ
: Correlation length determined by T–S fitting
*f*_a_
: Amphiphilicity factor determined by T–S fitting

## Data availability

The data are available in the ESI† or upon request from the authors.

## Author contributions

Di Wu: methodology, investigation, data analysis and writing – original draft. Vaibhav Dev: investigation. Valentin Bobrin: investigation, data analysis. Kenny Lee: data analysis and writing – review and editing. Cyrille Boyer: conceptualization, writing – review and editing, funding acquisition and supervision.

## Conflicts of interest

There are no conflicts to declare.

## Supplementary Material

SC-OLF-D4SC05597G-s001

SC-OLF-D4SC05597G-s002
