# Supplementary material for: Nanostructure design of 3D printed materials through macromolecular architecture
Source: Chem Sci. 2024 Nov 1;15(46):19345–58. doi: 10.1039/d4sc05597g (PMC11533054; doi:10.1039/d4sc05597g)
Supplement: SC-015-D4SC05597G-s001 [file SC-015-D4SC05597G-s001.pdf]

Supplementary Information (SI) for Chemical Science.
This journal is © The Royal Society of Chemistry 2024

Supporting Information

Nanostructure design of 3D printed materials through branched macromolecular architecture

Di Wu[1], Vaibhav Dev[1], Valentin A. Bobrin[1], Kenny Lee[1], and Cyrille Boyer[1, 2*]

[1]Cluster for Advanced Macromolecular Design, School of Chemical Engineering, University of New South Wales, Sydney, NSW 2052 (Australia)

[2]Australian Centre for Nanomedicine, School of Chemical Engineering, University of New South Wales, Sydney, NSW 2052, Australia

Email: cboyer@unsw.edu.au

Materials

Unless otherwise stated, all chemicals were used as received. The solvents were of either HPLC or AR grade; these included acetonitrile (MeCN, RCI Labscan Limited, RCI Premium), *N,N*-dimethylacetamide (DMAc, RCI Labscan Limited, HPLC), methanol (Chem-supply, 99.9%), dichloromethane (DCM, Supelco, ≥99%), and anhydrous dichloromethane (DCM, Sigma-Aldrich, ≥99.8%). Aluminium oxide basic (Acros Organics, Brockmann I, 50–200 µm, 60A), magnesium sulfate (62.0-70.0% w/w as $MgSO_4$, Chem-supply, LR), 4-(dimethylamino) pyridine (DMAP, Aldrich, ≥99%), 1-ethyl-3-(3-dimethylaminopropyl)carbodiimide hydrochloride (EDC-HCl, AmBeed, 99%), 2-(*n*-butylthiocarbonothioylthio) propanoic acid (BTPA, Boron Molecular, >95%), diphenyl (2,4,6-trimethylbenzoyl) phosphine oxide (TPO, Sigma-Aldrich, >97%), 2,2'-azobis(2-methylpropionitrile) solution (AIBN solution, Sigma-Aldrich, 0.2 M in toluene), acrylic acid (AA, anhydrous, Sigma-Aldrich, 99%) and poly(ethylene glycol) diacrylate average M_n = 250 (PEGDA, Sigma-Aldrich, >92%) were used as received. *n*-butyl acrylate (BA, Sigma-Aldrich, ≥99%) and 2-hydroxyethyl acrylate (HEA, Sigma-Aldrich, ≥99%) were passed through a basic aluminium oxide column to remove inhibitor prior to use.

Methods

Nuclear magnetic resonance (NMR)

All NMR spectra were recorded on Bruker Avance III 400 MHz spectrometer using an external lock ($CDCl_3$).

Size exclusion chromatography (SEC)

Analysis of the molecular weight distributions of the polymers were determined using a Shimadzu modular system composed of an SIL-20A auto-injector, a Polymer Laboratories 5.0 µm bead-size guard column (50 × 7.5 mm^2) followed by three linear PL (Styragel) columns (10^5, 10^4 and 10^3), an RID-10A differential refractive-index (RI) detector, and a UV detector. The eluent was DMAc (containing 0.03% w/v LiBr and 0.05% w/v 2,6-dibutyl-4-methylphenol (BHT)) at 50 °C, run at a flow rate of 1.0 mL/min. The SEC was calibrated using narrow polystyrene (PSTY) standards with molecular weights of 200 – 10^6 g/mol.

Attenuated total reflectance - Fourier transform infrared (ATR-FTIR) spectroscopy

ATR-FTIR spectroscopy was performed to monitor photopolymerization kinetics using a Bruker Alpha FTIR spectrometer equipped with room temperature DTGS detectors. After taking a background reading of the empty plate, 20 µL of polymerization resin was pipetted onto the ATR crystal plate. An absorption spectrum was then obtained by scanning the droplet from 400-4000 cm^{-1}. After an initial reading, the droplet was irradiated with a Thorlabs mounted LED with a collimation adapter (λ_{max} = 405 nm, I_0 = 2 mW cm^{-2}) and subsequently the IR absorption spectra were obtained at various times to determine the integral of the vinylic peak at time t_x. Vinyl bond conversions were calculated from the disappearance of the C=C stretching peak at 1630 cm^{-1} normalized to the C=O stretching peak at 1760 cm^{-1} as an internal standard using **Equation S1**:

$$Conversion\,(\%) = 100 \times \left(1 - \frac{\frac{int_x}{std_x}}{\frac{int_0}{std_0}}\right) \quad \textbf{(S1)}$$

Where int_x is the integral of the 1600-1650 cm^{-1} peak at x min of irradiation, std_x is the integral of the 1670-1800 cm^{-1} peak at x min of irradiation, int_0 is the initial integral of the 1600-1650 cm^{-1} peak before irradiation, and std_0 is the initial integral of the 1670-1800 cm^{-1} peak before irradiation. The vinyl bond conversion was monitored using 15 s intervals between 0 to 2 min. (Please note that the resin denoted as 12-100-43.9 (**Table S2**) was measured with 30 s intervals between 0 to 6 min.)

Atomic force microscopy (AFM)

All AFM measurements were performed on the Bruker Dimension ICON SPM, with a Nanoscope V controller (software version 9.70). An OTESPA-R3 probe (from Bruker AFM probes) was used to perform the tapping mode measurements. Mechanical properties measurements were performed using peak force tapping mode on a top layer of printed object using the SCANASYST probe (from Bruker AFM probes). The scan size was set to 1 µm and 300 nm. The scan rate was set at around 0.6 to 0.7 Hz with a peakforce of approximately 500 pN. The feedback gain was adjusted accordingly to optimize tracking of the specimen surface, without any significant feedback noise. The resolution of the image was set to 512 pixels per line for 1 µm scan size and 256 samples/line for 300 nm scan size. For peakforce QNM measurements, the tip was calibrated using the thermal tunning method. AFM images were analyzed using NanoScope Analysis software, version 1.7. For the statistical length analysis, at least 50 particles were carefully traced by hand to determine average domain size and domain spacing using ImageJ software. Histograms of the size distribution were constructed. Average macroCTA domain width (D_m) and center-to-center domain spacing (d_{AFM}) were calculated using **Equation S2 and S3**:

$$D_m = \frac{\sum_{i=1}^{n} N_i d_i}{\sum_{i=1}^{n} N_i} \quad \textbf{(S2)}$$

$$d_{AFM} = \frac{\sum_{i=1}^{n} N_i d_i}{\sum_{i=1}^{n} N_i} \quad \textbf{(S3)}$$

Where N is the number of observations and d is the determined size for each measurement of D_m or d_{AFM}, which were defined according to **Figure S7**.

Small-angle X-ray scattering (SAXS)

SAXS experiments were performed on an Anton Paar SAXSPoint 2.0 system with a Cu K_α (λ = 0.154 nm) microfocus X-ray source (50 kV/1 mA) and Dectris Eiger 1M detector. Data was collected at room temperature, under vacuum for 5 min from a sample at a sample-to-detector distance of 0.575 m. Samples were 3D printed at the thickness of 2×100 µm layers. Data was reduced to 1D by radial averaging the 2D detector after converting pixel positions to $q = (4\pi/\lambda)\sin\theta$, where 2θ is the scattering angle). The domain spacing was calculated using **Equation S4**:

$$d_{SAXS} = \frac{2\pi}{q} \quad \textbf{(S4)}$$

SAXS fitting using Teubner-Strey (T-S) model[1]

The position and the sharpness of SAXS peaks of microphase-separated 3D printed materials were fitted using T-S model in SasView software. According to T-S model (**Equation S5**)

$$I(q) = \frac{1}{a_2 + c_1 q^2 + c_2 q^4} + b \quad \textbf{(S5)}$$

Where $q = (4\pi/\lambda)\sin\theta$, λ is the wavelength, 2θ is the scattering angle; b is background scattering; a_2, c_1, c_2 are fitting parameters used to calculate domain spacing (d_{TS}), correlation length (ξ) and the amphiphilicity factor (f_a) using **Equations S6-S8** below:

$$d_{TS} = 2\pi[\frac{1}{2}\left(\frac{a_2}{c_2}\right)^{1/2} - \frac{1 c_1}{4 c_2}]^{-1/2} \quad \textbf{(S6)}$$

$$\xi = [\frac{1}{2}\left(\frac{a_2}{c_2}\right)^{\frac{1}{2}} + \frac{1 c_1}{4 c_2}]^{-1/2} \quad \textbf{(S7)}$$

$$f_a = \frac{c_1}{\sqrt{4 a_2 c_2}} \quad \textbf{(S8)}$$

Tensile testing

Dog-bone specimens were designed using Tinkercad 3D modelling software by modifying ASTM D638 Type I specimen[2] and the object was exported as an .stl file. Specimen dimensions were thickness (T) = 2.04 mm, width overall (WO) = 8.38 mm, length overall (LO) = 50.3 mm, distance between grips (D) = 36 mm, gauge length (G) = 15.79 mm, width at the center = 6 mm.

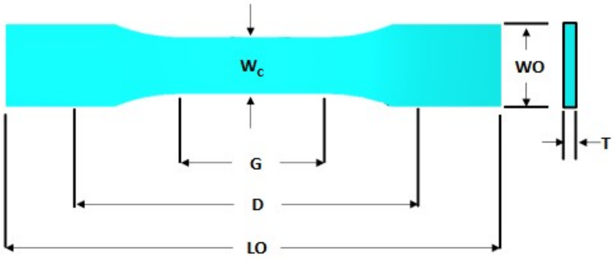

The mechanical tensile stress tests were performed using a Mark–10 ESM303 with a 1 kN force gauge model M5–200. The speed of testing was 1.1 mm/min. All tensile results were performed in at least triplicate. The tensile stress was calculated from the applied force divided by the initial cross-sectional area of the gage section. The strain was determined as the change in gage length relative to the original specimen gage length, expressed as a percentage. Toughness was determined by calculating the area under a stress-strain curve using the trapezoidal rule.

Swelling testing

3D printed square prism materials ($l \times w \times t = 8 \times 8 \times 2$ mm) were examined to determine swelling properties in water and toluene. For each resin formulation, the swelling study was performed in duplicates. For a typical procedure, the squre prisms were weighed before swelling to provide W_0. They were then immersed in 5 mL of water or toluene in 20 mL glass vials for swelling. At fixed time intervals, the samples were weighed after removing the excess solvent on surface by wiping with paper towel to get W_t. The swelling ratios by weight changes were then calculated by the following **Equation S9**:

$$Swelling\ ratio\ (wt\%) = 100 \times \frac{W_t - W_0}{W_0} \tag{S9}$$

Differential scanning calorimetry

The glass transition temperature was acquired on Q20 differential scanning calorimeter produced by TA Instruments. The temperature range was from -20 to 100 °C, with a heating rate of 10 °C per minute.

Estimation of $\chi_{\text{P(AA-stat-PEGDA)-b-PBA}}$ by group molar contribution method[3]

Note S1: $\chi_{\text{P(AA-stat-PEGDA)-b-PBA}}$ was estimated using **Equation S10**:

$$\chi_{\text{P(AA-}stat\text{-PEGDA)-}b\text{-PBA}} = (1 - x)\ \chi_{\text{PEGDA–PBA}} + x\ \chi_{\text{PAA-PBA}} + x\ (1 - x)\ \chi_{\text{PAA–PEGDA}} \tag{S10}$$

where x is the weight fraction of AA in P(AA-*stat*-PEGDA) block ($x = 0.54$). χ_{12} was calculated using **Equation S11**:

$$\chi_{12} = \frac{VN_A}{RT}(\delta_1 - \delta_2)^2 \tag{S11}$$

where V is the reference volume (set to 118 Å^3), R is the gas constant (1.987 cal mol^{-1} K^{-1}), T is temperature (set to 298 K), N_A is the Avogadro's number (6.02×10^{23} mol^{-1}), δ ((cal cm^{-3})$^{1/2}$) is solubility parameter estimated using the group molar contribution method proposed by Small (**Equation S12**):

$$\delta = \frac{d\sum G}{M} \tag{S12}$$

where d (g cm^{-3}) is density, M is monomer molecular weight, $\sum G$ is the sum of the molar attraction constants. Estimated δ values were as follows: δ_{PBA} = 9.15 $cal^{1/2}$ $cm^{-3/2}$, δ_{PAA} = 11.18 $cal^{1/2}$ $cm^{-3/2}$, δ_{PEGDA} = 8.50 $cal^{1/2}$ $cm^{-3/2}$. Then, χ parameters were calculated using Equation (S10): $\chi_{PEGDA\text{-}PBA}$ = 0.051, $\chi_{PAA\text{-}PBA}$ = 0.497, $\chi_{PAA\text{-}PEGDA}$ = 0.865. Subsequently, $\chi_{P(AA\text{-}stat\text{-}PEGDA)\text{-}b\text{-}PBA}$ was calculated using Equation (S10): $\chi_{P(AA\text{-}stat\text{-}PEGDA)\text{-}b\text{-}PBA}$ = 0.505.

3D printing setup and procedure

A typical procedure for fabricating 3D printed objects is as follows: A 3D object was designed using Tinkercad 3D modelling software and the object was exported as an .stl file format. The .stl file was opened using Photon Workshop, where the Z lift speed was set to 3 mm/s and Z retract speed was set to 2 mm/s, while the Z lift distance was set to 6 mm. Printing parameters, such as layer thickness and exposure time, were defined in Photon workshop, sliced, and exported as .pws files for 3D printing. The .pws file copied to a flash drive for use with a masked LCD 3D printer (Anycubic Photon Mono SE) with a violet light LED array (λ_{max} = 405 nm, I_0 = 2 mWcm^{-2}). All samples were 3D printed using a layer thickness of 100 μm, 6 s off time and 1 bottom layer. The cure time per layer was 60 s for all the 3D printed samples except for the lantern model (40 s). After 3D printing was completed, the printed objects were separated from a build plate, washed with ethanol and post-cured under violet light (λ_{max} = 405 nm, I_0 = 10 mWcm^{-2}) for 30 min.

Synthetic Procedure

A: RAFT polymerization of *n*-butyl acrylate and 2-hydroxyethyl acrylate in acetonitrile

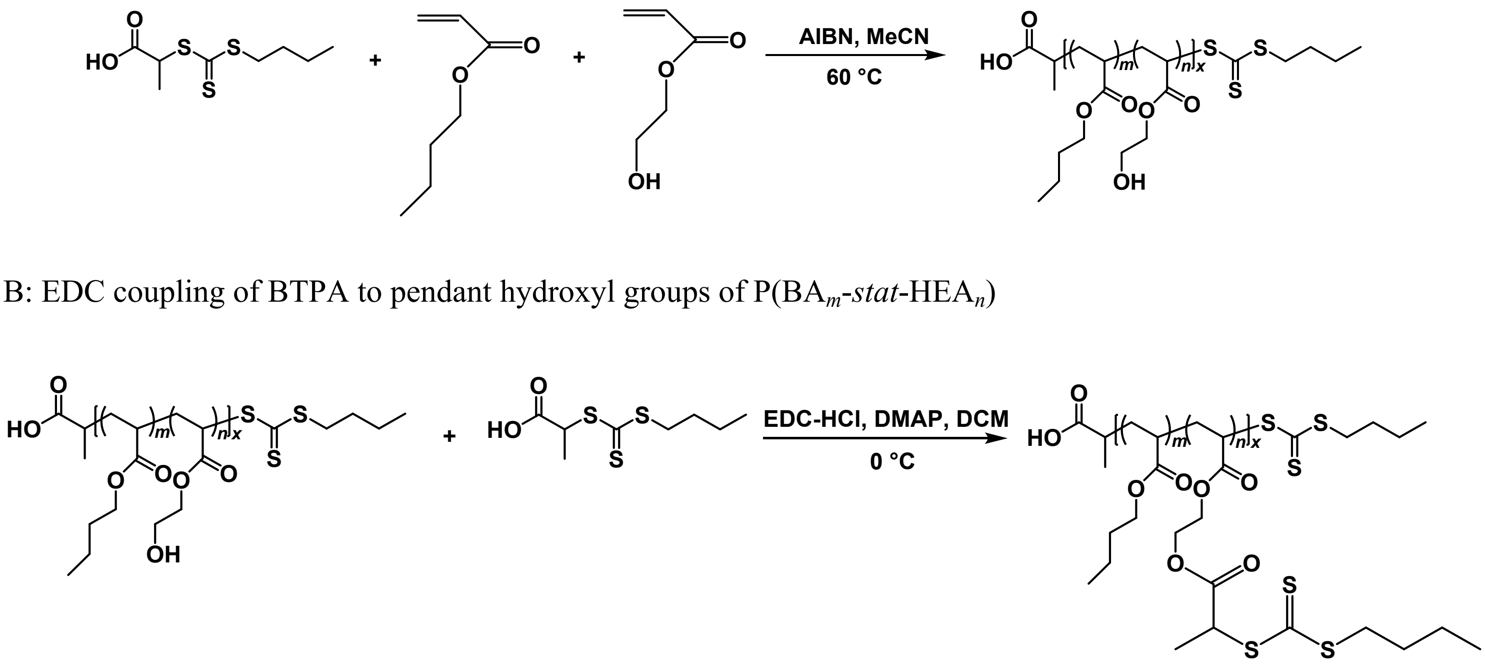

Figure S1. Two-step synthesis of branched macroCTAs: A) RAFT polymerization and of *n*-butyl acrylate and 2-hydroxyethyl acrylate and B) EDC coupling of BTPA to pendant hydroxyl groups of P(BA$_m$-*stat*-HEA$_n$).

Protocol for synthesis of macroCTA (describe for 3-200 as an example, Table S1)

A: RAFT polymerization of n-butyl acrylate and 2-hydroxyethyl acrylate

n-butyl acrylate (25 g, 0.195 mol), 2-hydroxyethyl acrylate (0.327 g, 2.81×10^{-3} mol), BTPA RAFT agent (0.224 g, 9.38×10^{-4} mol) and AIBN (0.2 M solution in toluene, 0.704 mL, 1.41×10^{-4} mol) were dissolved in acetonitrile (50 mL). The mixture was deoxygenated by purging with nitrogen for 60 min, and then polymerized for 18 h at 60 °C. The reaction was stopped by exposure to air. The polymer solution was concentrated by rotary evaporation to obtain P(BA$_m$-*stat*-HEA$_n$)-CTA.

B: EDC coupling of BTPA to pendant hydroxyl groups of P(BA$_m$-*stat*-HEA$_n$)

P(BA$_m$-*stat*-HEA$_n$) (22.3 g, 8.69×10^{-4} mol), BTPA RAFT agent (0.864 g, 3.63×10^{-3} mol), 4-

(dimethylamino) pyridine (0.0443 g, 3.63×10^{-4} mol) were dissolved in anhydrous dichloromethane (50 mL) according to pre-calculated ratios. The mixture was deoxygenated by purging with nitrogen for 60 min and then placed in an ice-water bath. EDC-HCl (0.695 g, 3.63×10^{-3} mol) in pre-determined quantity was dissolved in anhydrous dichloromethane (50 mL) and deoxygenated by purging with nitrogen for 30 min in an ice-water bath. After that, EDC-HCl solution was added dropwise into polymer solution in an ice-water bath. The reaction was carried out for 18 h with the first hour being in the ice-water bath.

The product was purified by washing the reaction mixture with Milli-Q water for 3 times (50 mL). The DCM phase was then dried over anhydrous $MgSO_4$, filtered, and concentrated by rotary evaporation. The polymer was recovered by precipitation into large excess of methanol, isolated by centrifugation. The fresh portion of methanol was added to the polymer residual and the resulting mixture was vortexed and centrifuged to isolate the polymer. The washing-centrifugation cycle was repeated 3 times. Then, the isolated polymer was dissolved in DCM (50 mL) and concentrated by rotary evaporation.

Using the same protocol, other macroCTAs were synthesized and the experimental conditions were summarized in **Table S1**.

Table S1. Experimental molar ratio between components in synthesis of branched macroCTAs.

macroCTA	[BA]/[HEA]/[BTPA]/[AIBN]	[P(BA-*stat*-HEA)]/[BTPA]/EDC-HCl]/[DMAP]
3-200	208/3/1/0.15	1/1.5/1.5/0.15
6-200	208/6/1/0.15	1/1.5/1.5/0.15
3-100	104/3/1/0.15	1/1.5/1.5/0.15
6-100	100/7/1/0.15	1/1.5/1.5/0.15
12-100	95/12/1/0.15	1/1.5/1.5/0.15

Additional data

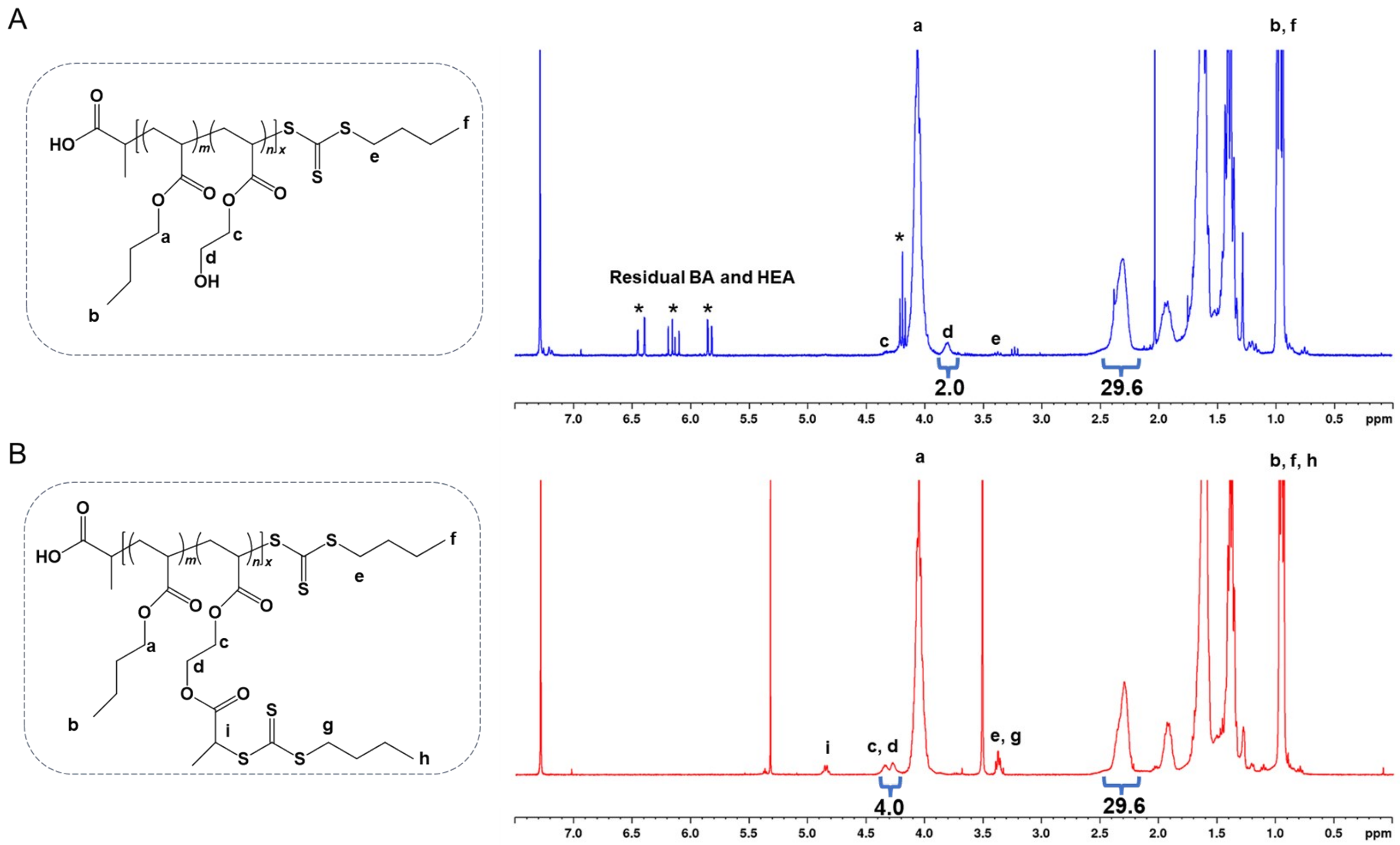

Figure S2. ^1H NMR spectra (400 MHz, $CDCl_3$, 298 K) of 3-100 macroCTA before (A) and after (B) EDC coupling. The spectra normalized by a resonance at 4.03 ppm. The peak labeled d in the 3-100 macroCTA corresponding to the CH_2 α- to the hydroxy group occurs at 3.8 ppm, which was shifted to 4.3 ppm after EDC coupling, now corresponding to the CH_2 α- to the new ester group. The complete shift of peak d indicates successful esterification with a reaction efficiency close to 100%.

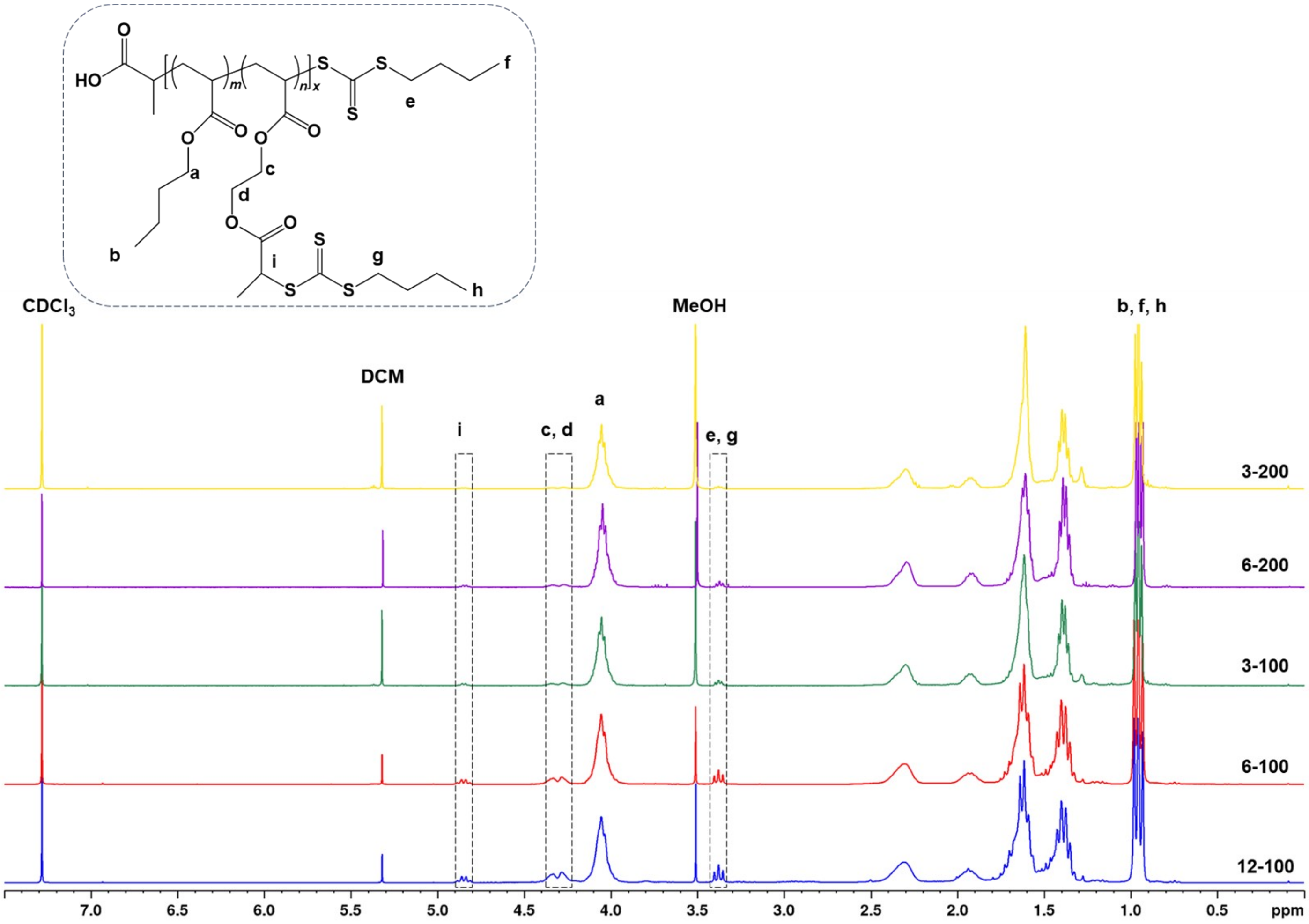

Figure S3. ^{1}H NMR spectra (400 MHz, $CDCl_3$, 298 K) of target macroCTAs with pendant CTA groups. The spectra normalized by a resonance at 4.03 ppm.

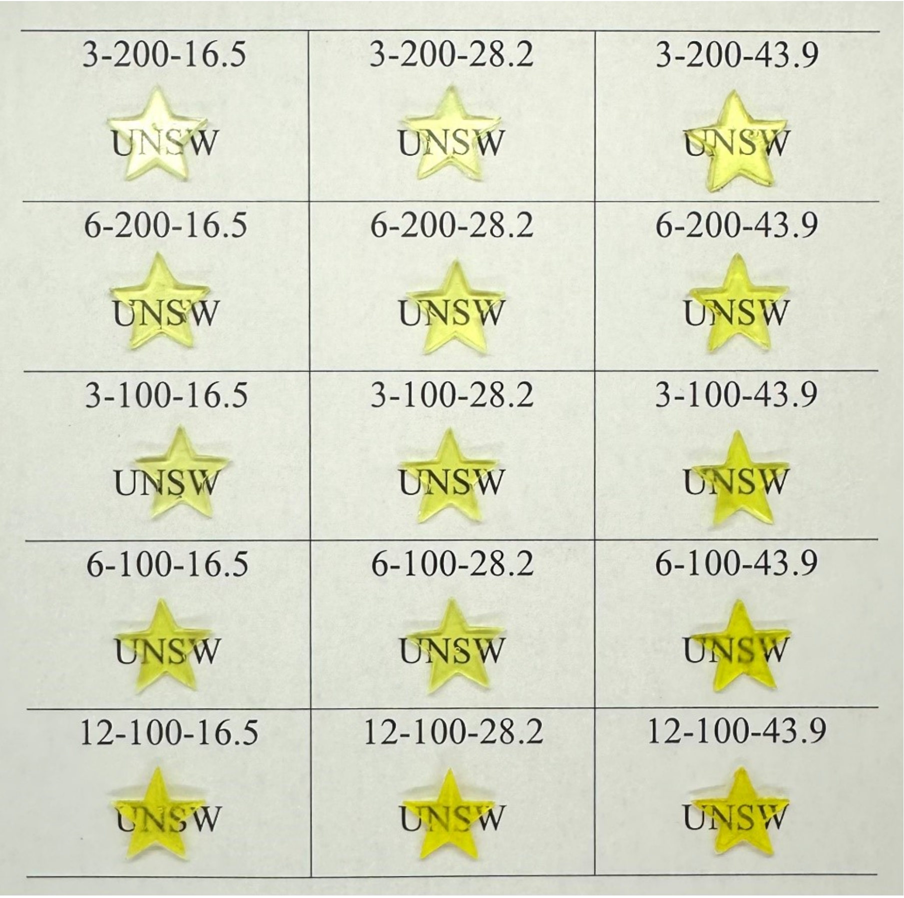

Figure S4. Star-shaped 3D printed objects using 15 formulations presented in **Table S2**.

Table S2. Molar ratios of [AA]/[PEGDA]/[macroCTA] for various resin formulations at three loading of macroCTA (16.5, 28.2 and 43.9 wt%). The molar ratio of [AA]/[PEGDA] was fixed at 4/1.

Wt% of macroCTA	macroCTA	X_p[a]	n+1[b]	Molar ratio between resin components			N_p[c]	χN_p[d]
				AA	PEGDA	macroCTA		
16.5	3-200	50	4	984	245	1	357	180
	6-200	28	7	988	256	1	204	103
	3-100	25	4	513	128	1	185	94
	6-100	14	7	545	136	1	112	56
	12-100	8	13	596	149	1	65	33
28.2	3-200	50	4	493	123	1	204	103
	6-200	28	7	495	124	1	116	59
	3-100	25	4	257	64	1	106	53
	6-100	14	7	273	68	1	63	32
	12-100	8	13	299	75	1	37	19
43.9	3-200	50	4	245	61	1	126	64
	6-200	28	7	246	62	1	72	36
	3-100	25	4	128	32	1	63	33
	6-100	14	7	136	34	1	39	20
	12-100	8	13	149	37	1	22	11

[a] – X_p is the number of repeating BA or HEA units per RAFT group, calculated from **Equation 1** (in main text); [b] – n_{pCTA}+1 is the total number of CTA groups per macroCTA chain, including pendant CTA groups and the terminal CTA gaup; [c] – N_p is the average total degree of polymerization of each RAFT agent, defined by **Equation 2** (in main text); [d] – Estimated χ = 0.505 from **Note S1**.

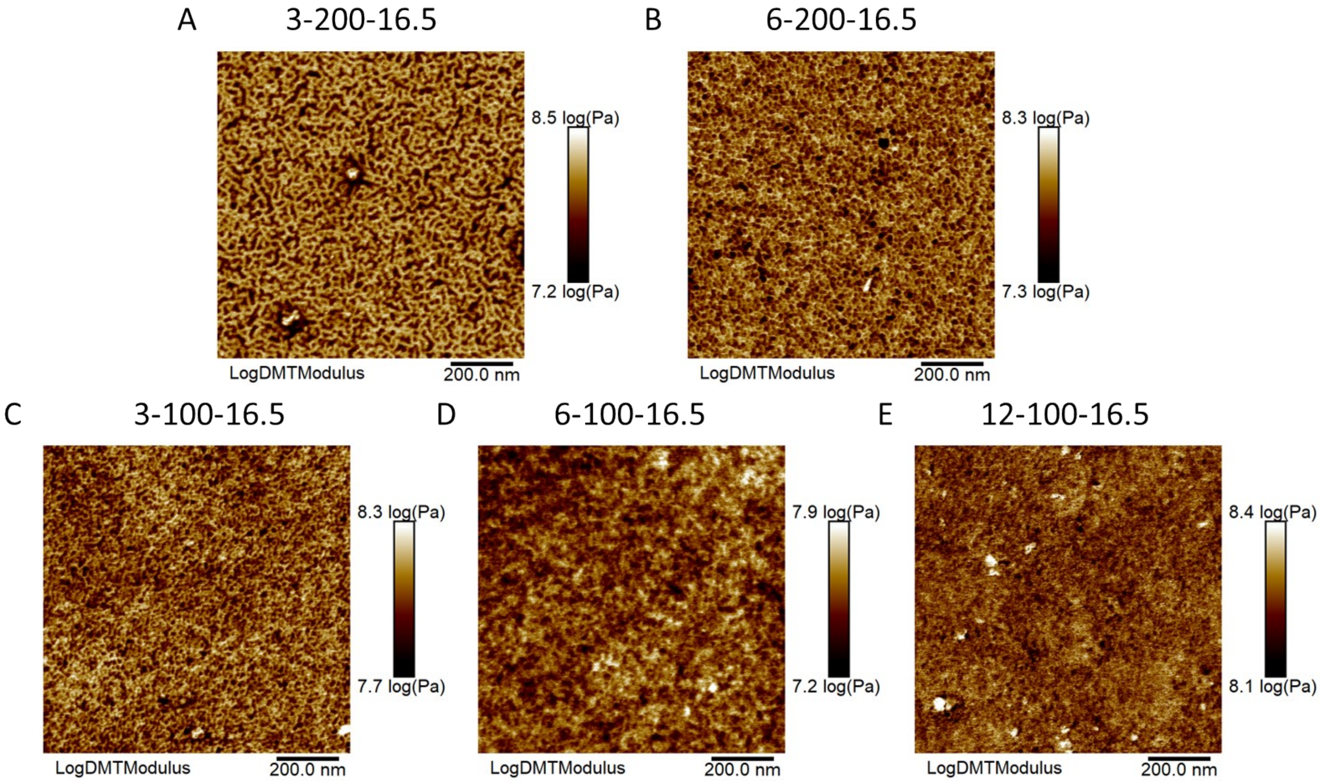

Figure S5. PeakForce QNM modulus map images of samples 3D printed using 16.5 wt% loading of various branched macroCTAs: A) 3-200; B) 6-200; C) 3-100; D) 6-100; E) 12-100. Materials were 3D printed using a molar ratio of [AA]/[PEGDA] = 4/1.

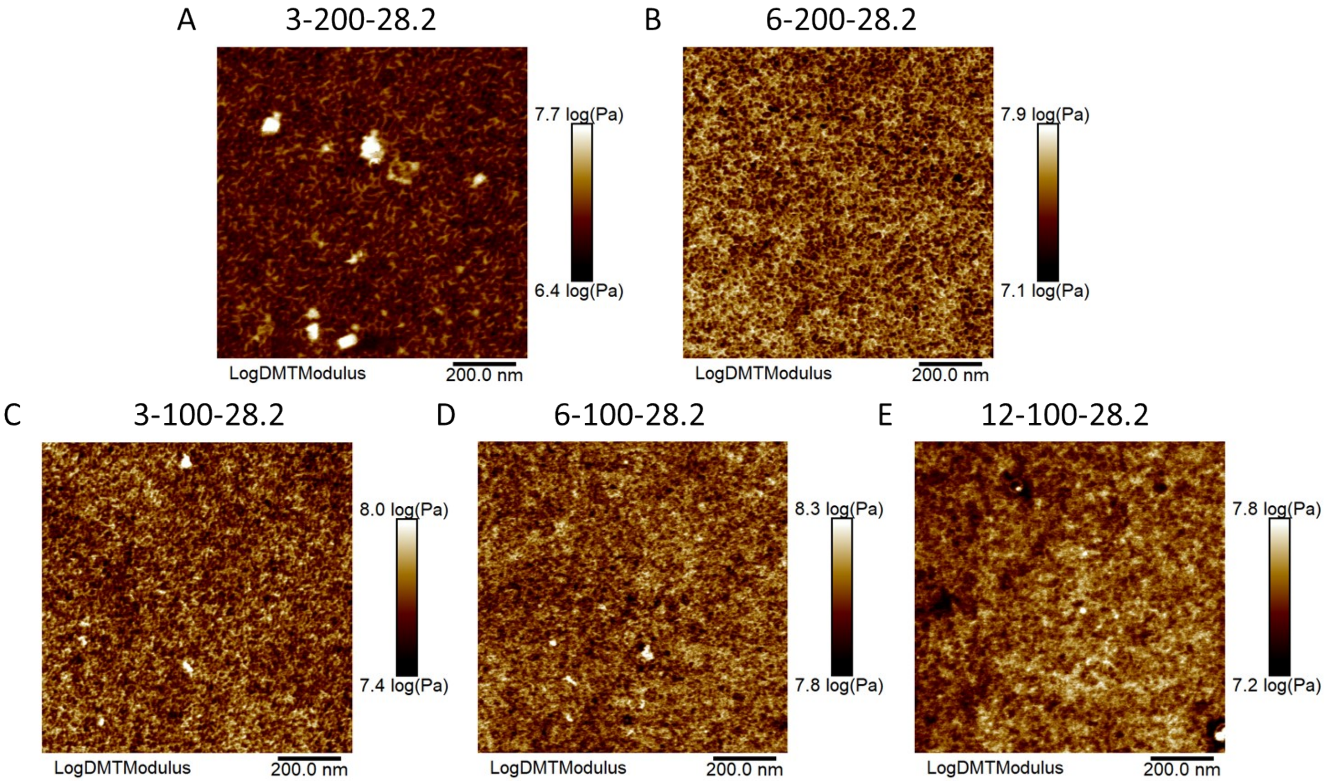

Figure S6. PeakForce QNM modulus map images of samples 3D printed using 28.2 wt% loading of various branched macroCTAs: A) 3-200; B) 6-200; C) 3-100; D) 6-100; E) 12-100. Materials were 3D printed using a molar ratio of [AA]/[PEGDA] = 4/1.

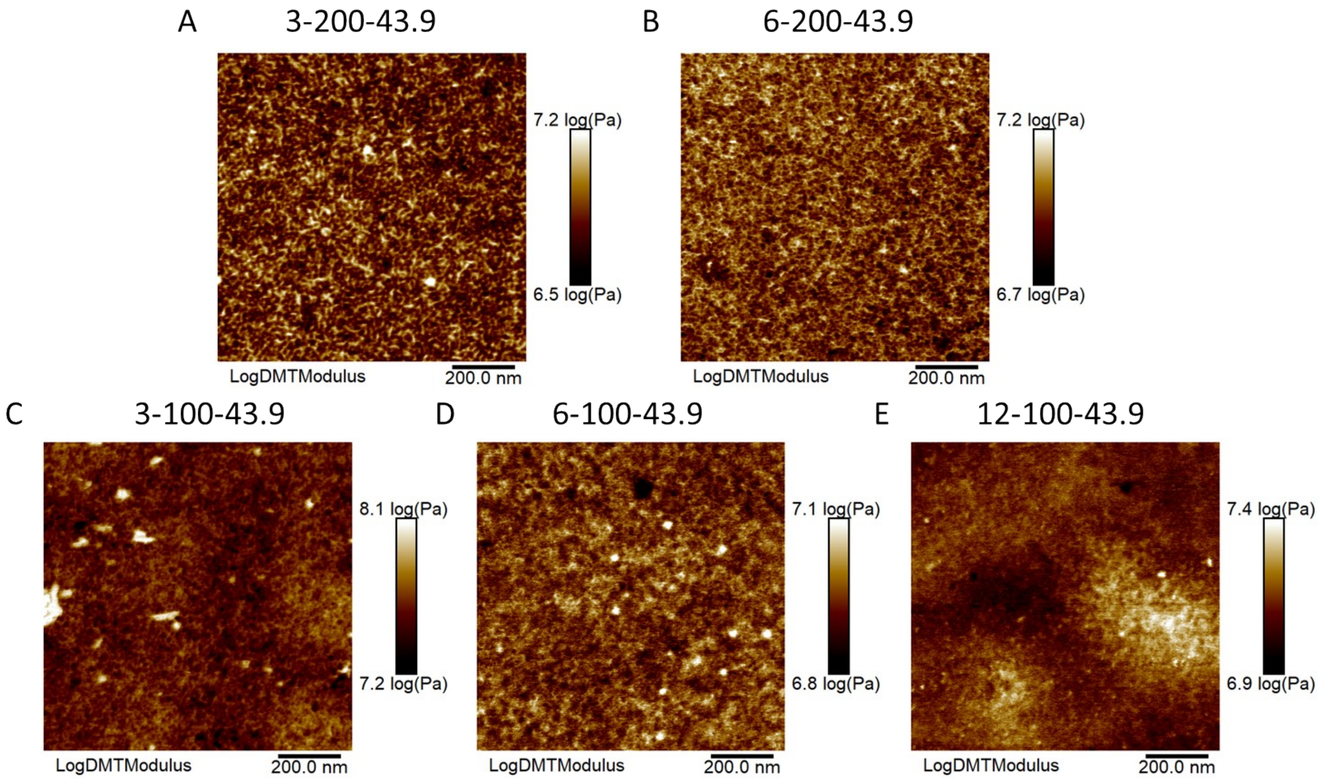

Figure S7. PeakForce QNM modulus map images of samples 3D printed using 43.9 wt% loading of various branched macroCTAs: A) 3-200; B) 6-200; C) 3-100; D) 6-100; E) 12-100. Materials were 3D printed using a molar ratio of [AA]/[PEGDA] = 4/1.

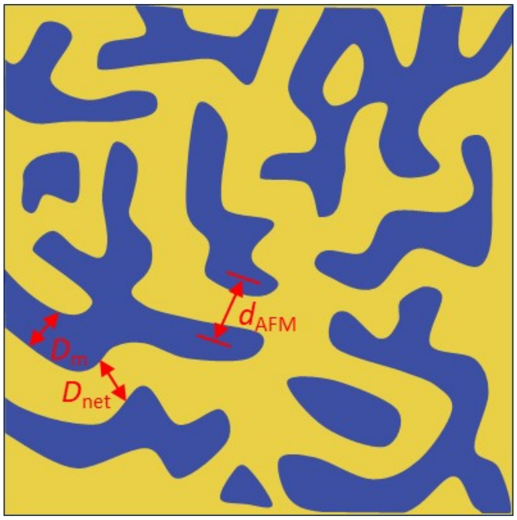

Figure S8. Schematic illustration of microphase-separated morphology. MacroCTA domains are shown in blue; *net*-P(AA-*stat*-PEGDA) domains are shown in yellow. D_m − macroCTA domain width; d_{AFM} – domain spacing; D_{net}. − *net*-P(AA-*stat*-PEGDA) domain width, which is $d_{AFM} - D_m$.

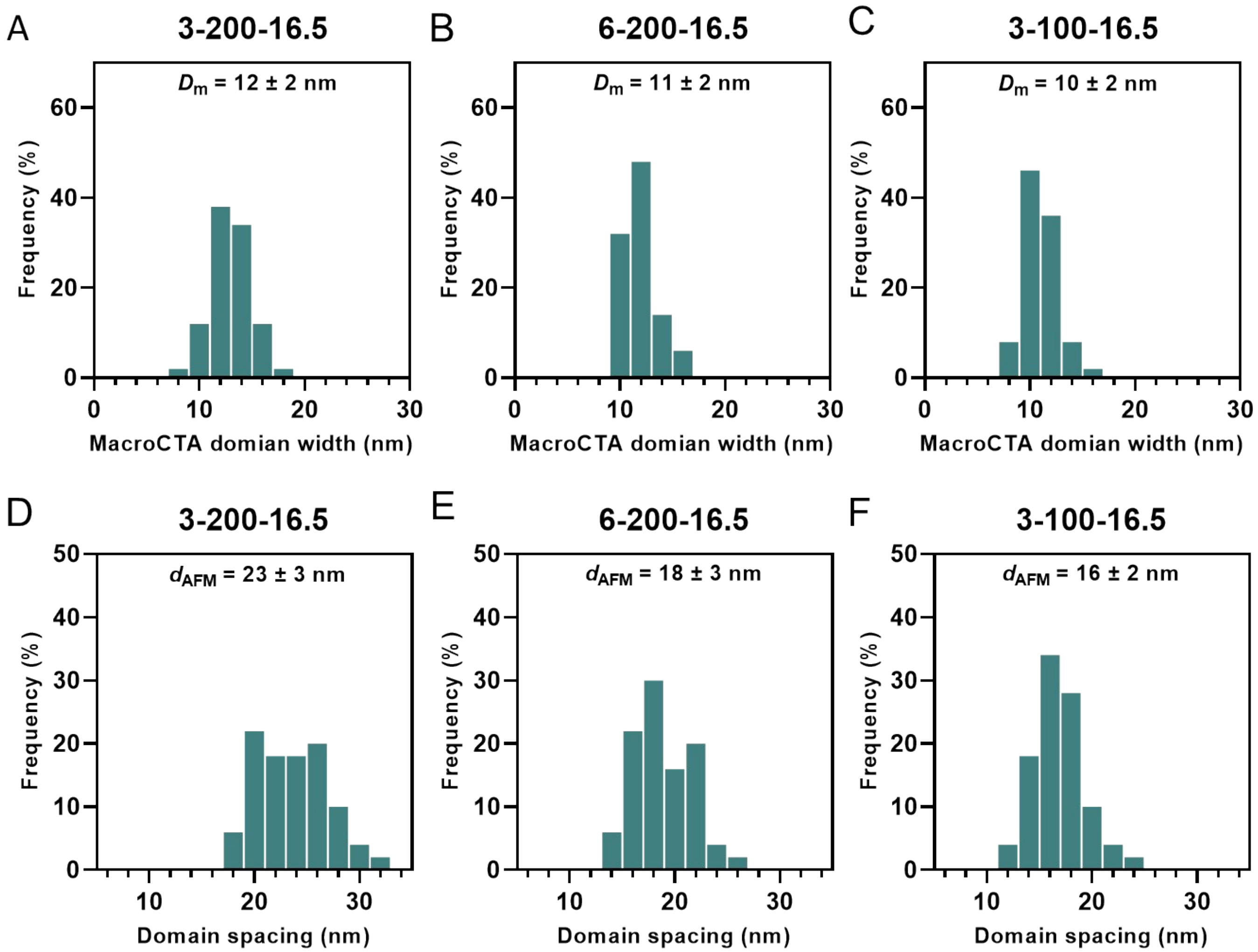

Figure S9. Average macroCTA domain width and domain spacing for materials 3D printed using various branched macroCTAs. A-C) MacroCTA domain width (D_m); D-F) Domain spacing (d_{AFM}). Materials were 3D printed using a molar ratio of [AA]/[PEGDA] = 4/1 at 16.5 wt% loading of macroCTA. Results of 6-100-16.5 and 12-100-16.5 were not reported due to the difficulty in precise measurements.

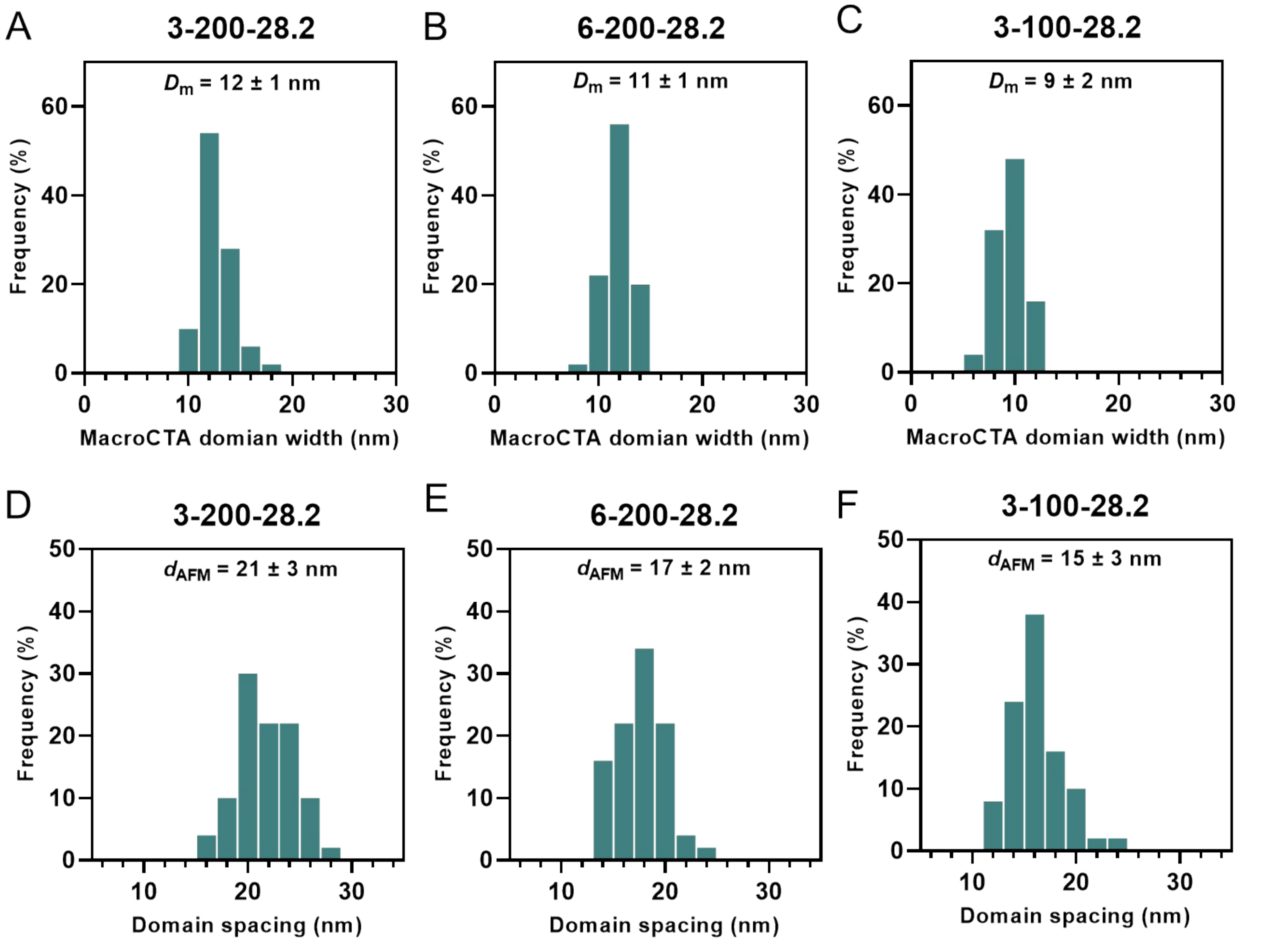

Figure S10. Average macroCTA domain width and domain spacing for materials 3D printed using various branched macroCTAs. A-C) MacroCTA domain width (D_m); D-F) Domain spacing (d_{AFM}). Materials were 3D printed using a molar ratio of [AA]/[PEGDA] = 4/1 at 28.2 wt% loading of macroCTA. Results of 6-100-28.2 and 12-100-28.2 were not reported due to the difficulty in precise measurements.

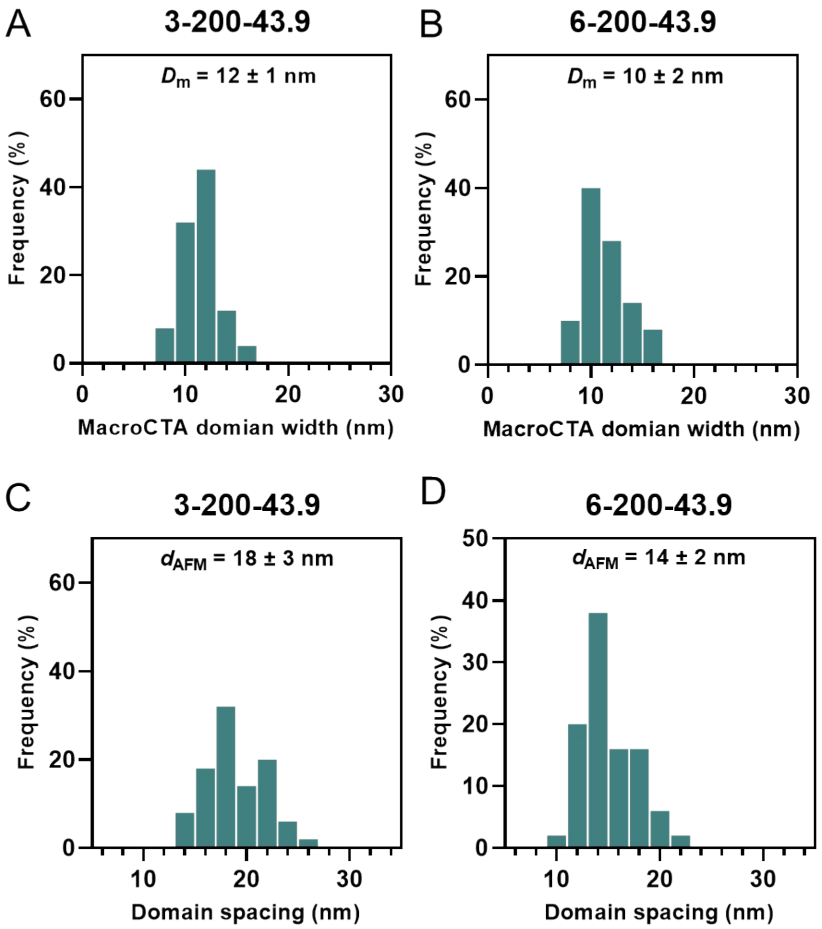

Figure S11. Average macroCTA domain width and domain spacing for materials 3D printed using various branched macroCTAs. A-B) MacroCTA domain width (D_m); C-D) Domain spacing (d_{AFM}). Materials were 3D printed using a molar ratio of [AA]/[PEGDA] = 4/1 at 43.9 wt% loading of macroCTA. Results of 3-100-43.9, 6-100-43.9 and 12-100-43.9 were not reported due to the difficulty in precise measurements.

Table S3. Parameter values obtained from fitting of SAXS peaks using Teubner-Strey (T-S) model, full width at 60% maximum of SAXS peaks and Porod exponent.

wt% of macroCTA	macro CTA	a_2[a]	c_1[a]	c_2[a]	d_{SAXS} (nm)[b]	d_{TS} (nm)[c]	ξ (nm)[d]	ξ/d_{TS}[e]	f_a[f]	Full width at 60% maximum[g]	Porod exponent[h]
16.5	3-200	91.5	-1198.2	6274.2	21.2	19.1	8.9	0.47	-0.79	0.174	3.6
	6-200	57.9	-303.5	731.2	14.4	12.7	5.2	0.41	-0.74	0.258	3.5
	3-100	82.1	-382.0	731.2	13.2	11.5	5.2	0.45	-0.78	0.288	3.5
	6-100	46.7	-87.0	81.0	8.6	7.8	3.0	0.38	-0.71	0.658	3.1
	12-100	27.5	-25.9	16.0	7.1	6.1	2.0	0.33	-0.62	0.886	2.4
28.2	3-200	113.3	-1400.3	6561.0	19.4	18.2	9	0.49	-0.81	0.184	3.8
	6-200	104.5	-572.5	1211.7	13.2	12.2	5.9	0.48	-0.80	0.277	3.8
	3-100	142.7	-692.3	1211.7	12.1	11.2	5.9	0.53	-0.83	0.288	3.8
	6-100	82.9	-164.3	133.6	8.2	7.5	3.4	0.45	-0.78	0.500	3.4
	12-100	46.2	-50.7	28.0	6.9	6	2.3	0.38	-0.71	0.745	3.0
43.9	3-200	120.5	-1267.0	4978.7	16.9	16.7	8.4	0.50	-0.82	0.217	3.8
	6-200	94.4	-466.8	915.1	12.4	11.7	5.5	0.47	-0.79	0.299	3.7
	3-100	130.4	-569.9	915.1	11.0	10.7	5.5	0.51	-0.82	0.321	3.7
	6-100	73.1	-160.4	150.1	8.4	8	3.5	0.44	-0.77	0.473	3.4
	12-100	42.0	-43.4	23.4	6.5	5.9	2.2	0.37	-0.69	0.777	2.7

[a] – parameters calculated from SAXS fitting using T-S model; [b] – domain spacing determined from SAXS; [c] – domain spacing determined from T-S fitting using **Equation S6**; [d] – correlation length determined from T-S fitting using **Equation S7**; [e] – The ratio of ξ/d_{TS} is a measure of the domain size polydispersity; the smaller the ratio, the larger the polydispersity[4]; [f] – amphiphilicity factor determined using **Equation S8**; [g] – the peak width in a function at 60% its maximum value (due to the asymmetry of the SAXS signals, the full width at half maximum (FWHM) is not directly applicable); [h] – the Porod

exponent describes how the scattered intensity I behaves as a function of the scattering vector q at large q values (high scattering angles).

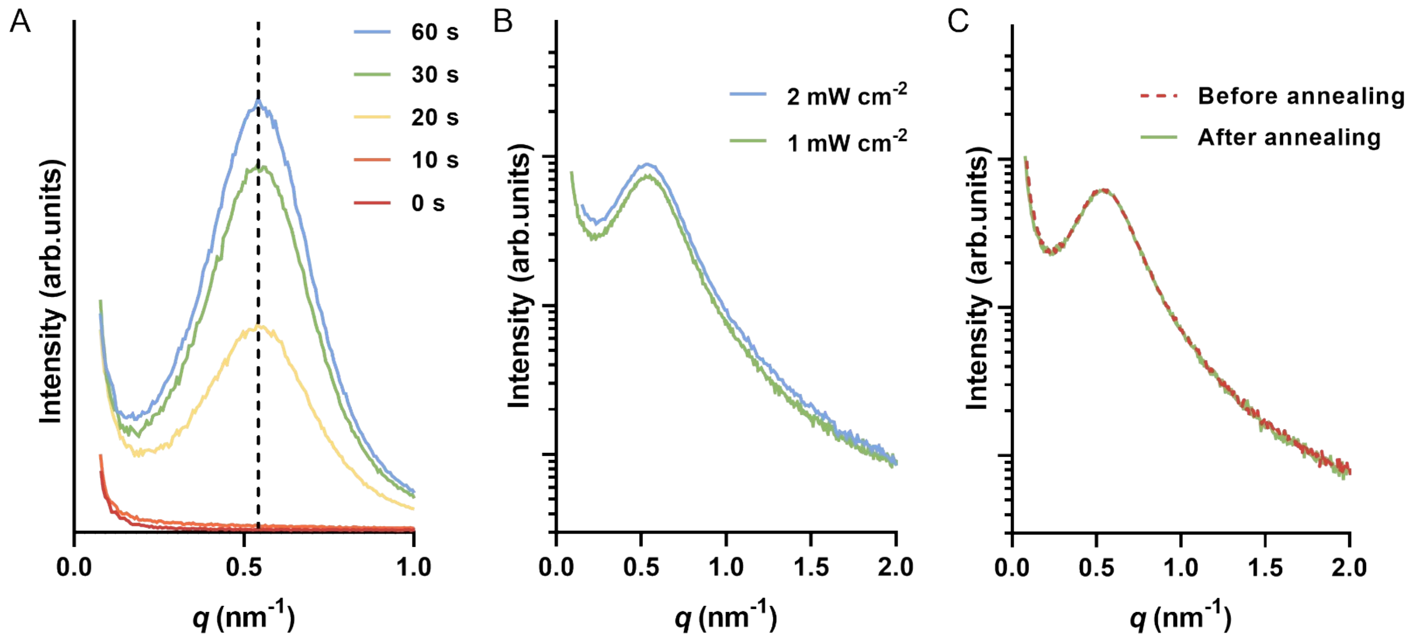

Figure S12. A) SAXS profiles of 3-100-28.2 resins irradiated under violet light (405 nm, 2 mW cm^{-2}) at different time points (0, 10, 20 30 and 60 s); B) 3D cured under violet light (405 nm) in different irradiation intensities (1 and 2 mW cm^{-2}); C) SAXS profiles of 3D printed 3-100-28.2 samples before and after annealing.

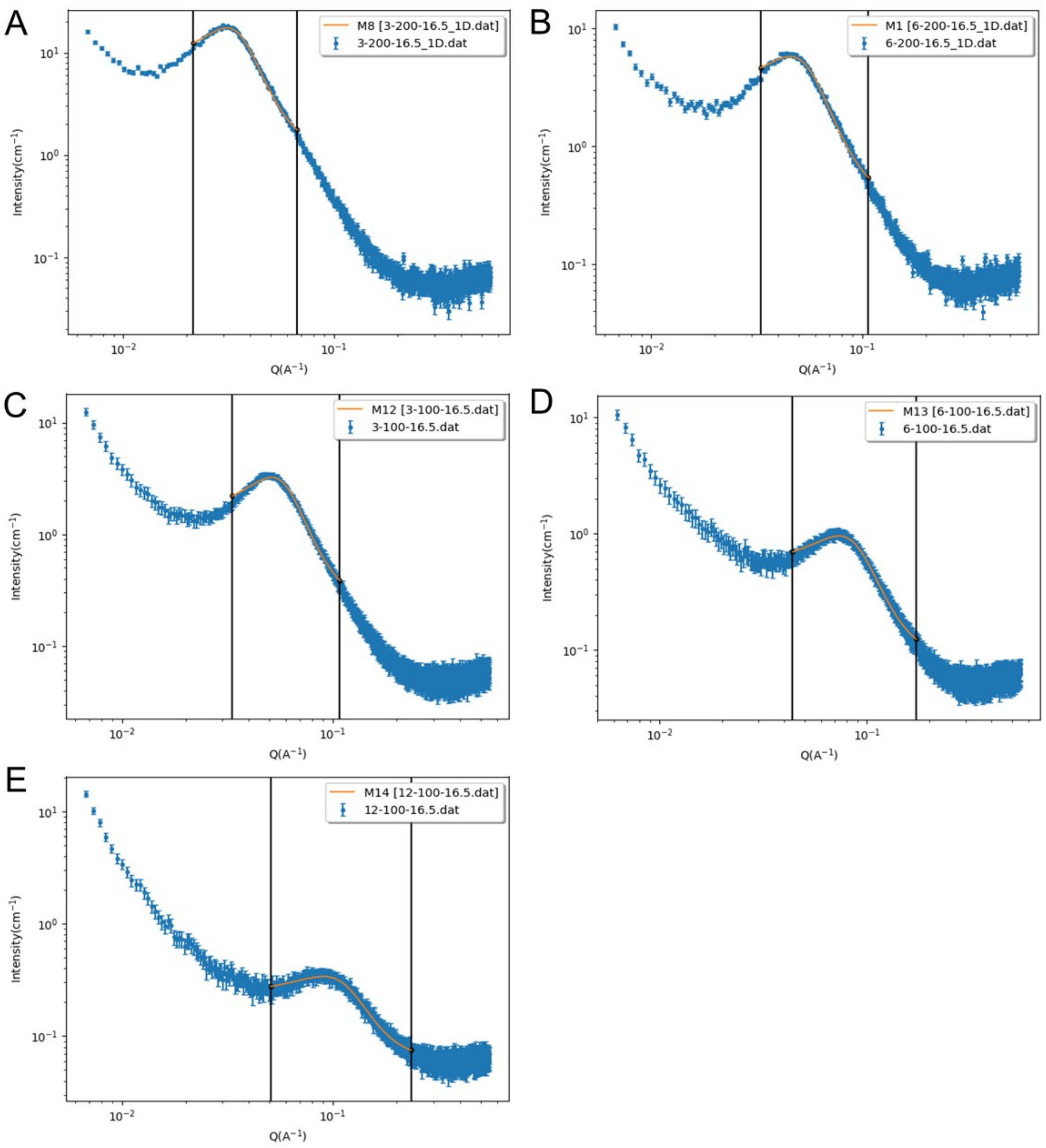

Figure S13. SAXS peaks fitted using Teubner-Strey (T-S) model for samples printed using 16.5 wt% macroCTA. A) 3-200; B) 6-200; C) 3-100; D) 6-100; E) 12-100. The SAXS scattering data is presented as a blue curve and T-S fit is presented as an orange curve.

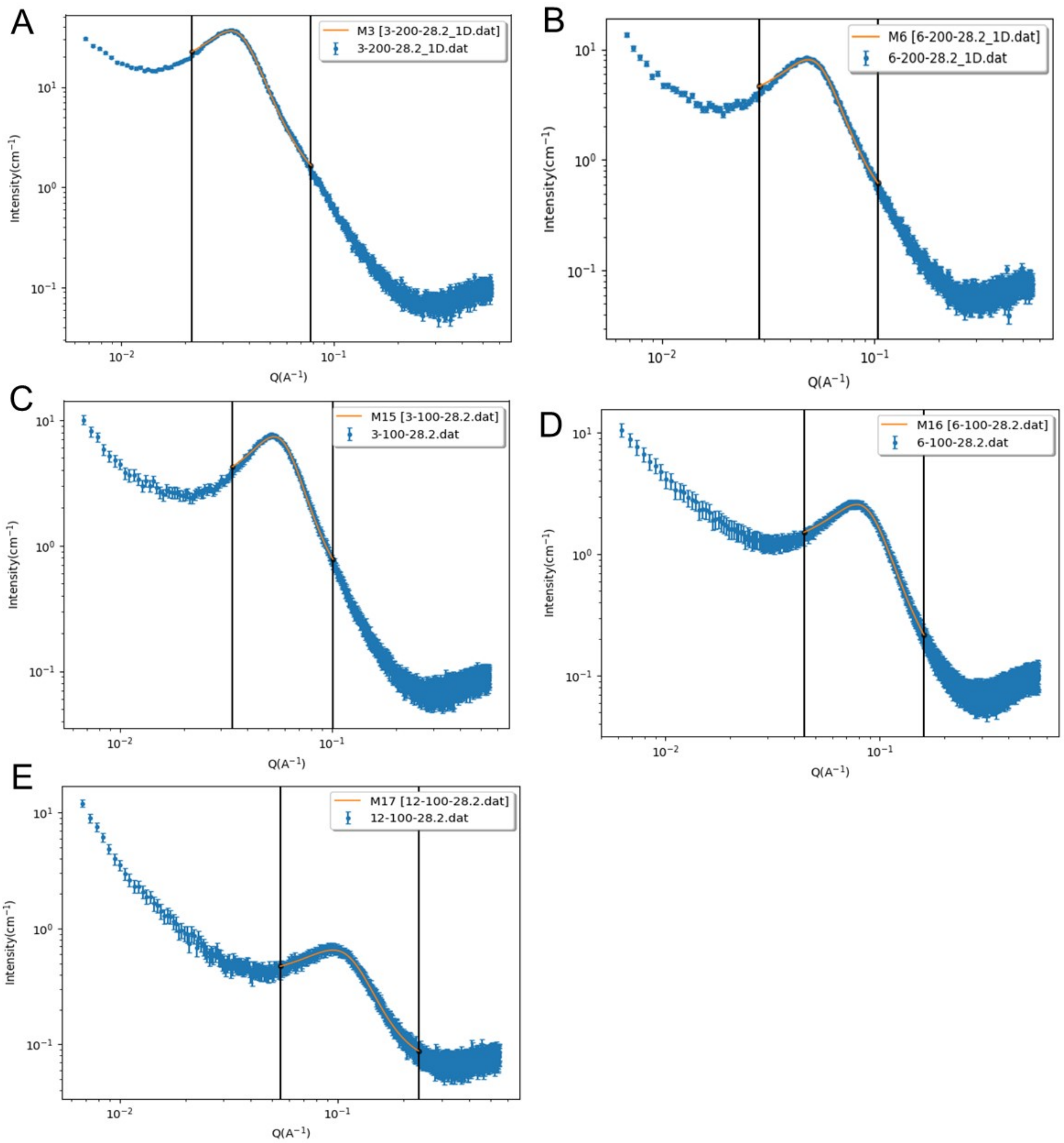

Figure S14. SAXS peaks fitted using Teubner-Strey (T-S) model for samples printed using 28.2 wt% macroCTA. A) 3-200; B) 6-200; C) 3-100; D) 6-100; E) 12-100. The SAXS scattering data is presented as a blue curve and T-S fit is presented as an orange curve.

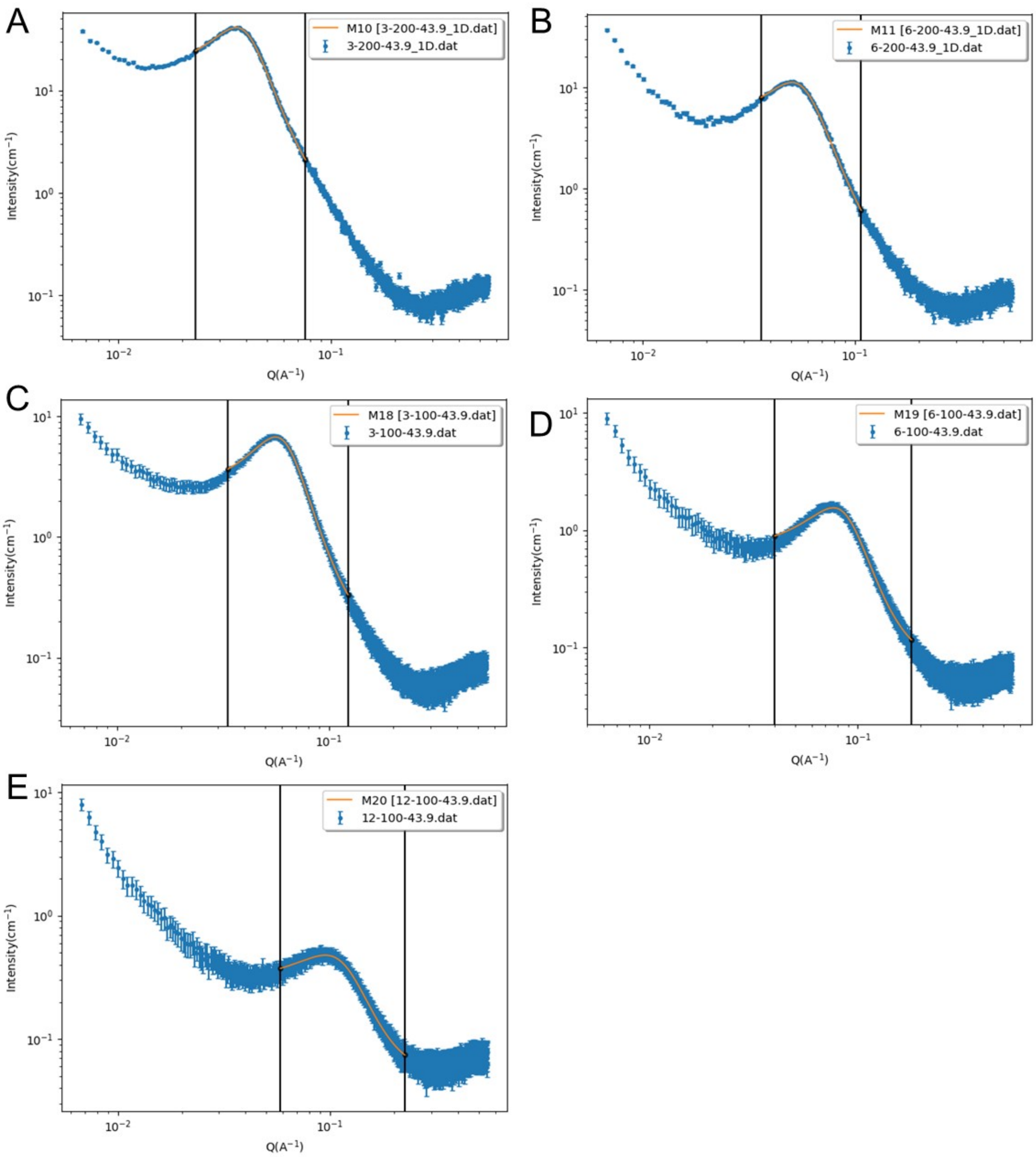

Figure S15. SAXS peaks fitted using Teubner-Strey (T-S) model for samples printed using 43.9 wt% macroCTA. A) 3-200; B) 6-200; C) 3-100; D) 6-100; E) 12-100. The SAXS scattering data is presented as a blue curve and T-S fit is presented as an orange curve.

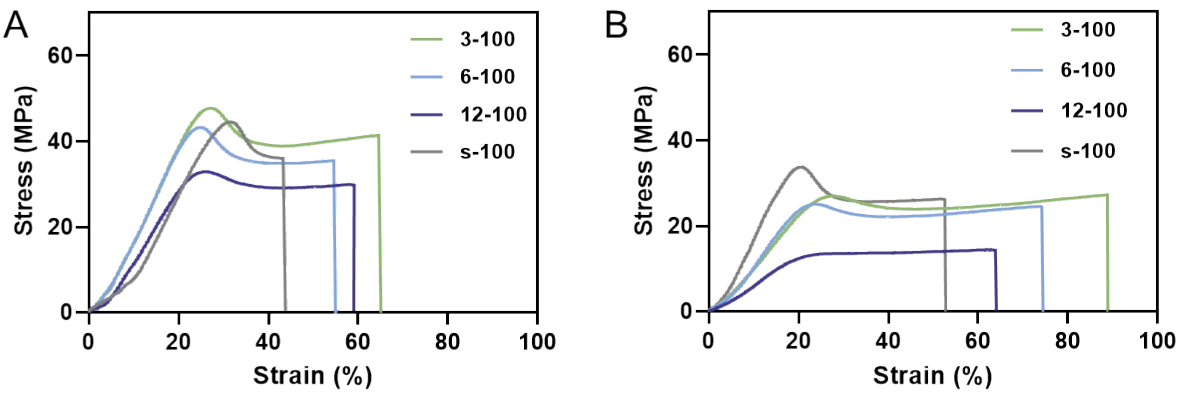

Figure S16. Representative stress–strain curves of 3D printed PIMS samples using various branched macroCTAs and 3D printed non-PIMS samples at two different weight percentage of macroCTA or BA-BTPA mixture in a resin formulation: A) 16.5 wt.%; B) 28.2 wt%. Materials were 3D printed using a molar ratio of [AA]/[PEGDA] = 4/1.

Table S4. Formulations for non-PIMS resins. The molar ratio of [AA]/[PEGDA] was fixed at 4/1.

Molar ratio of [BA]/[BTPA]	Resin components (wt%)			
	BA-BTPA mixture	AA	PEGDA	TPO
100/1	16.5	44.5	38.5	0.5
	28.2	38.1	33.0	0.7

Table S5. Summary of mechanical properties for 3D printed PIMS materials using various branched macroCTAs and 3D printed non-PIMS materials at 16.5 or 28.2 wt% of macroCTA or BA-BTPA mixture in a resin formulation.

Wt% of macroCTA or BA-BTPA mixture	Type	Young's modulus (MPa)[a]	Stress at break (MPa)[a]	Elongation at break (%)[c]	Toughness (MJ m^{-3})[d]
16.5	3-100	225.0 ± 10.8	41.3 ± 0.5	62.7 ± 5.9	20.9 ± 2.5
	6-100	229.7 ± 4.2	37.4 ± 1.4	53.0 ± 5.3	15.9 ± 2.0
	12-100	163.0 ± 4.4	28.6 ± 1.1	61.3 ± 4.0	14.0 ± 0.8
	s-100[e]	222.7 ± 24.8	42.1 ± 5.3	43.7 ± 5.5	12.8 ± 2.7
28.2	3-100	133.3 ± 8.4	27.0 ± 0.8	88.0 ± 3.6	19.1 ± 1.3
	6-100	139.3 ± 5.5	25.3 ± 0.9	71.7 ± 5.8	14.6 ± 1.4
	12-100	67.3 ± 10.6	13.5 ± 0.8	68.7 ± 5.7	7.1 ± 0.4
	s-100[f]	212.7 ± 7.1	27.2 ± 1.1	52.3 ± 7.0	12.4 ± 2.1

[a] – Young's Modulus is defined by the ratio between stress (force per unit area) and strain (proportional deformation) in the linear elasticity regime of a uniaxial deformation. [b] – Tensile stress at break was reported as the maximum tensile strength immediately before break. [c] – Elongation at break was reported as the maximum elongation of the sample immediately before break. [d] – Toughness was determined by calculating the area under a stress-strain curve using the trapezoidal rule. [e] – s-100 is statistical terpolymer with the formulation of [BA]/[BTPA]/[AA]/[PEGDA] = 100/1/489/122 (0.5 wt% of TPO). [f] – s-100 is statistical terpolymer with the formulation of [BA]/[BTPA]/[AA]/[PEGDA] = 100/1/245/61 (0.7 wt% of TPO). The data represents the mean ± s.d. of at least three independent experiments for each material.

Table S6. Formulations for statistical copolymer simulating interfacial layer. The molar ratio of [AA]/[PEGDA] was fixed at 4/1.

Resin components	Molar ratio	Mass fraction (wt%)
BA	40	51.4
AA + PEGDA	40	43.1
BTPA	1	4.8
TPO	\	0.7

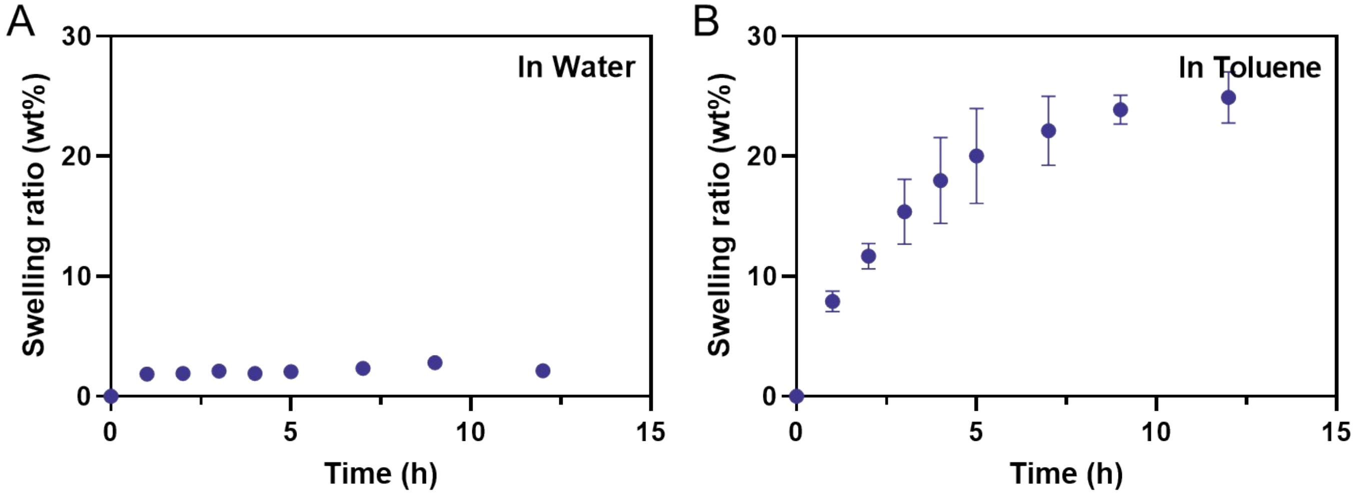

Figure S17. Swelling ratio (wt%) of 3D printed statistical copolymer materials using the mixture of BA, AA, PEGDA monomers with BTPA (the molar ratio of [BA]:[AA+PEGDA]:[BTPA] = 40:40:1) in different solvents. A) In water. B) In toluene. Error bars indicate standard deviation in duplicate measurements. Some error bars fall within the size of the markers.

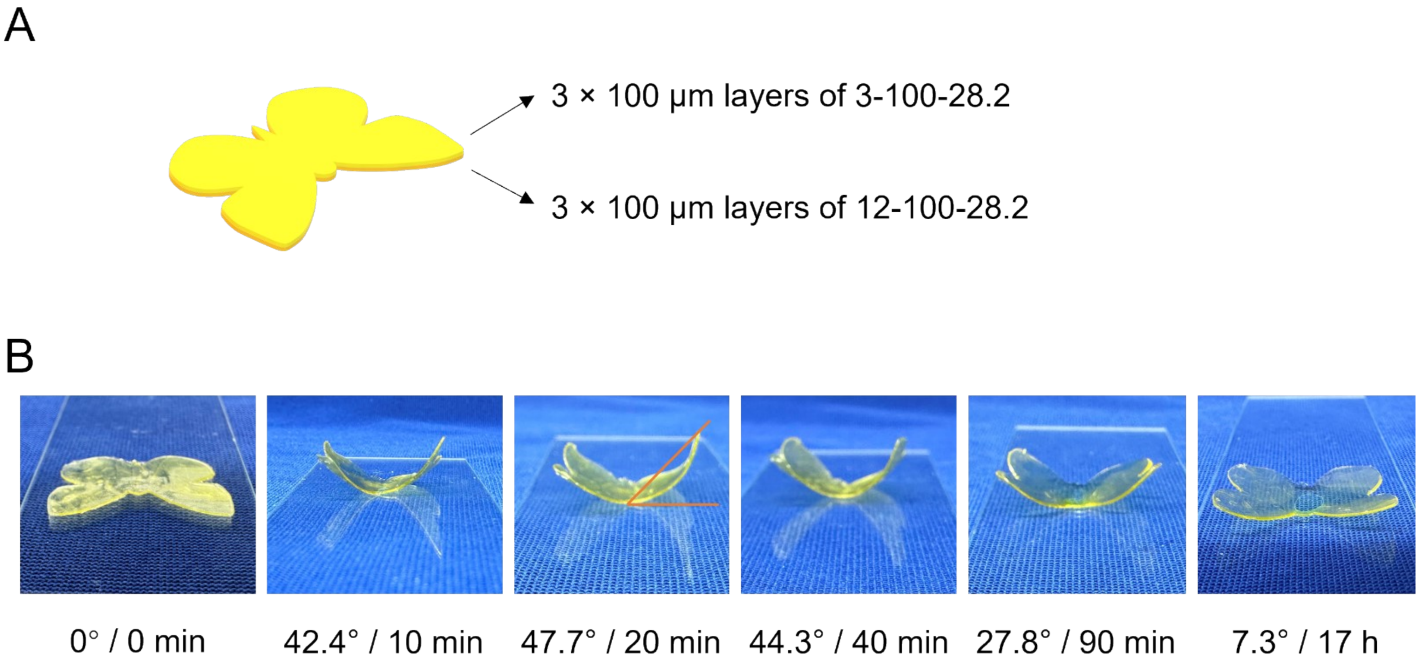

Figure S18. A) Digital model and fabrication details of multi-material butterfly: The top 3×100 μm layers of the butterfly were made using the resin of 3-100-28.2 while the bottom 3×100 μm layers of the butterfly were made using the resin of 12-100-28.2. B) Swelling-induced actuation in toluene of a multi-material butterfly 3D printed using two kinds of resins (The bending angle is measured from the highest point of the butterfly's right wing to the inclination angle formed with the glass plane from the front view).

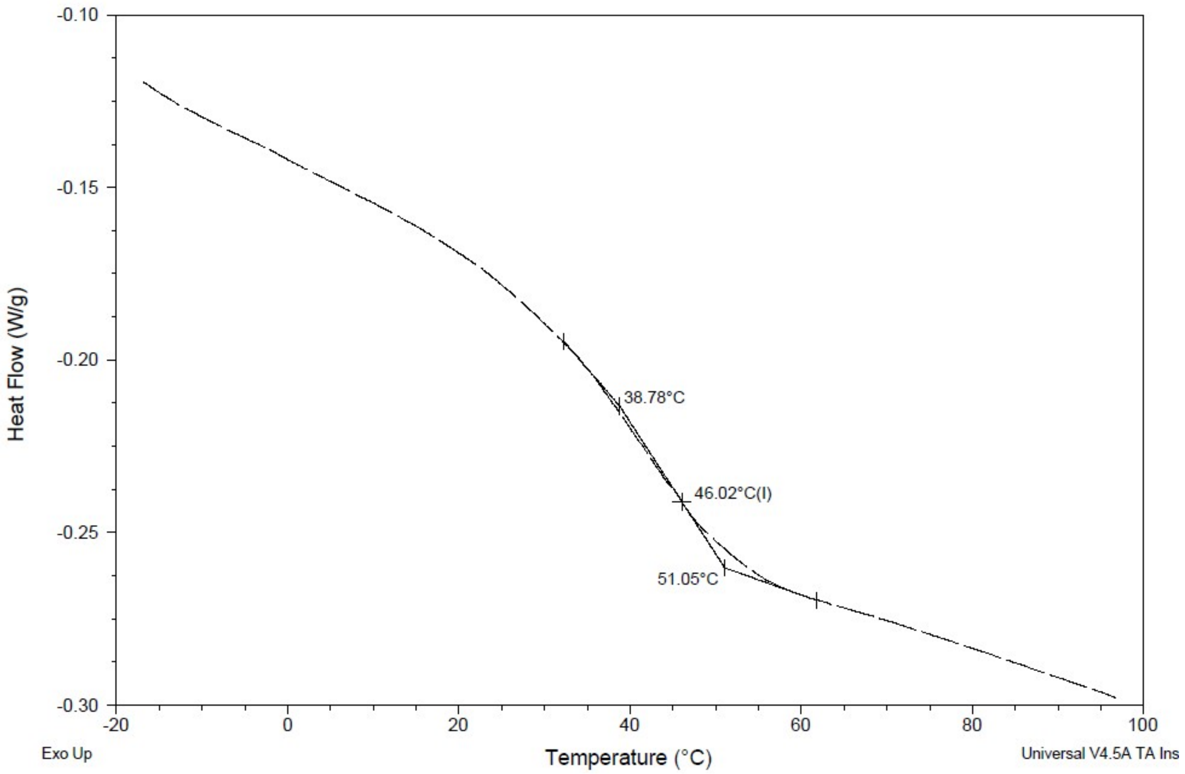

Figure S19. DSC curve of 3-100-28.2 sample.

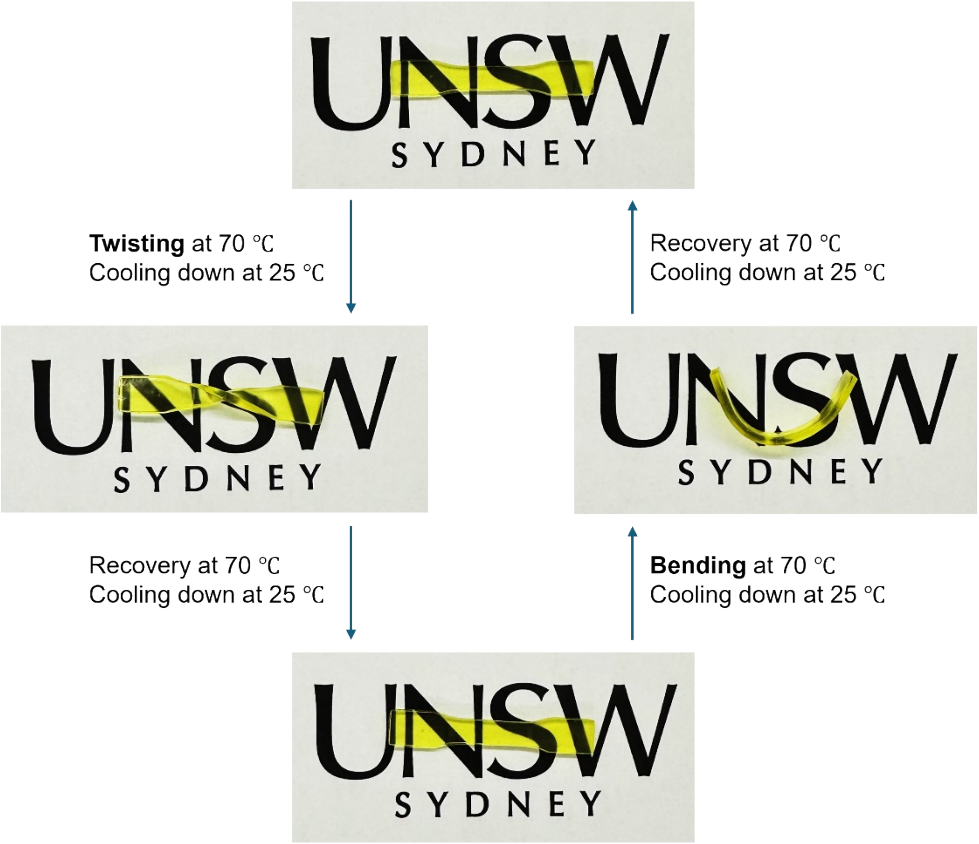

Figure S20. Shape memory behaviors of 3-100-28.2 sample. The sample was placed at 70 °C to soften, enabling to be deformed by force (bending or twisting). Subsequently, the sample was cooled down at 25 °C under mechanical stress to fix the deformation. Upon reheating to 70 °C, the sample recovered its original shape within one minute and kept it after cooling down (See **Video S1**).

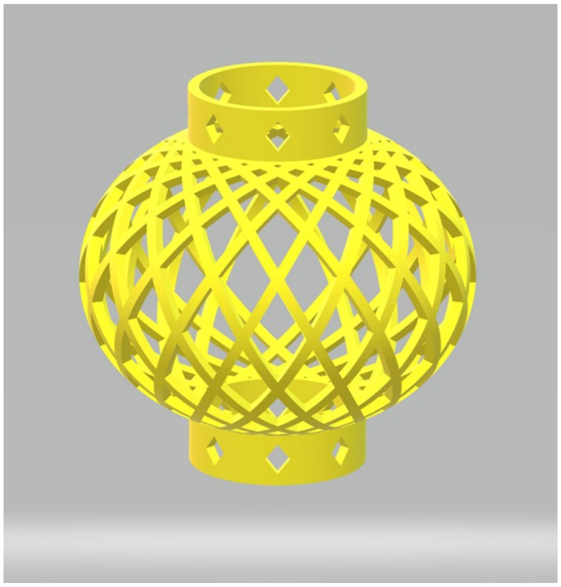

Figure S21. Digital model of Chinese lantern.

Supplementary References

(1) Teubner, M.; Strey, R. Origin of the scattering peak in microemulsions. *The Journal of Chemical Physics* **1987**, *87* (5), 3195-3200.

(2) International, A. *Standard test method for tensile properties of plastics*; ASTM international, 2014.

(3) Bobrin, V. A.; Yao, Y.; Shi, X.; Xiu, Y.; Zhang, J.; Corrigan, N.; Boyer, C. Nano-to macro-scale control of 3D printed materials via polymerization induced microphase separation. *Nature Communications* **2022**, *13* (1), 3577.

(4) Chen, S.; Chang, S.; Strey, R. On the interpretation of scattering peaks from bicontinuous microemulsions. *Trends in Colloid and Interface Science IV* **1990**, 30-35.
